# Characterization of *UGT716A1* as a Multi-substrate UDP:Flavonoid Glucosyltransferase Gene in *Ginkgo biloba*

**DOI:** 10.3389/fpls.2017.02085

**Published:** 2017-12-07

**Authors:** Xiaojia Su, Guoan Shen, Shaokang Di, Richard A. Dixon, Yongzhen Pang

**Affiliations:** ^1^Key Laboratory of Plant Resources and Beijing Botanical Garden, Institute of Botany, Chinese Academy of Sciences, Beijing, China; ^2^Institute of Animal Science, Chinese Academy of Agricultural Sciences, Beijing, China; ^3^University of Chinese Academy of Sciences, Beijing, China; ^4^BioDiscovery Institute and Department of Biological Sciences, University of North Texas, Denton TX, United States; ^5^Beijing Advanced Innovation Center for Tree Breeding by Molecular Design, Beijing Forestry University, Beijing, China

**Keywords:** *Ginkgo biloba*, flavonoids, UGT716A1, multi-substrate UGT, EGCG, flavanol gallate glycosides

## Abstract

*Ginkgo biloba* L., a “living fossil” and medicinal plant, is a well-known rich source of bioactive flavonoids. The molecular mechanism underlying the biosynthesis of flavonoid glucosides, the predominant flavonoids in *G. biloba*, remains unclear. To better understand flavonoid glucosylation in *G. biloba*, we generated a transcriptomic dataset of *G. biloba* leaf tissue by high-throughput RNA sequencing. We identified 25 putative UDP-glycosyltransferase (*UGT*) unigenes that are potentially involved in the flavonoid glycosylation. Among them, we successfully isolated and expressed eight *UGT* genes in *Escherichia coli*, and found that recombinant UGT716A1 protein was active toward broad range of flavonoid/phenylpropanoid substrates. In particular, we discovered the first recombinant UGT protein, UGT716A1 from *G. biloba*, possessing unique activity toward flavanol gallates that have been extensively documented to have significant bioactivity relating to human health. *UGT716A1* expression level paralleled the flavonoid distribution pattern in *G. biloba*. Ectopic over-expression of *UGT716A1* in *Arabidopsis thaliana* led to increased accumulation of several flavonol glucosides. Identification and comparison of the *in vitro* enzymatic activity of UGT716A1 homologs revealed a UGT from the primitive land species *Physcomitrella patens* also showed broader substrate spectrum than those from higher plants *A. thaliana, Vitis vinifera*, and *Medicago truncatula*. The characterization of *UGT716A1* from *G. biloba* bridges a gap in the evolutionary history of *UGTs* in gymnosperms. We also discuss the implication of *UGT716A1* for biosynthesis, evolution, and bioengineering of diverse glucosylated flavonoids.

## Introduction

*Ginkgo biloba* has existed on earth for 200 million years and is called a “living fossil”. *G. biloba* has been recorded in clinical practice for more than four centuries, since the Ming Dynasty, in the Compendium of Materia Medica (Li and Luo, 2004). *G. biloba* leaf tissue accumulates abundant secondary metabolites, including flavonoids and terpenoids (van Beek, 2002; van Beek and Montoro, 2009). EGb761, the standardized extract of *G. biloba* leaf, contains about 24% flavonol glycosides, 20% non-flavonol glycosides, 7% proanthocyanidins, 2% flavanols, and 6% terpenoids (van Beek and Montoro, 2009). EGb761 is widely used as a dietary supplement or phytomedicine in western countries, and has been applied in clinical therapy to treat cardiovascular and neurological disorders, such as Alzheimer’s disease (Nash and Shah, 2015). EGb761 possess many benefits for human health, such as radical scavenging and antioxidant activities (DeFeudis and Drieu, 2000), anti-inflammation activity (Kotakadi et al., 2008), antiapoptotic activity (Serrano-García et al., 2013), and neuroprotective activity (Bastianetto et al., 2000). Recently, more than 60 different flavonoids have been identified in *G. biloba*, and the majority of them are glycosylated (Liu et al., 2015).

**FIGURE 1:**
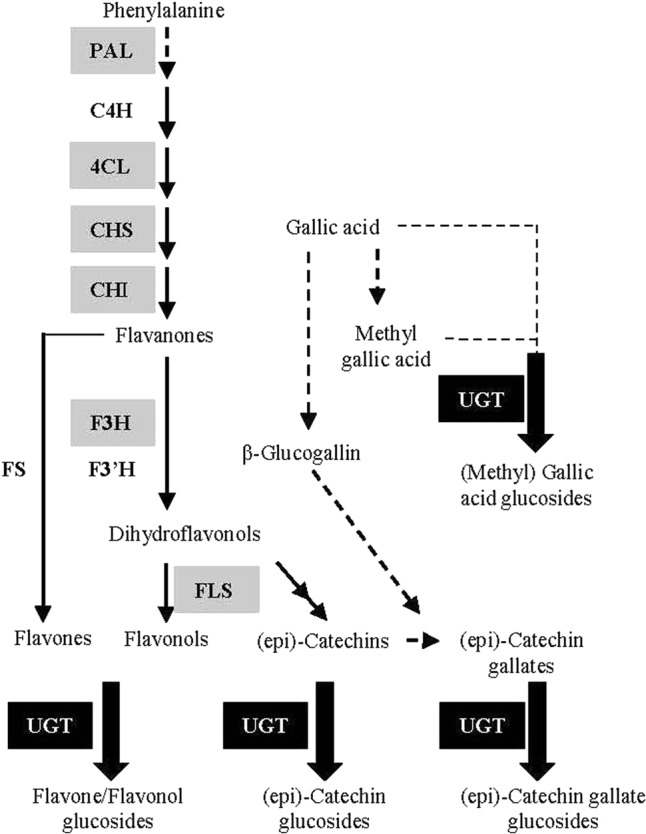
Schematic representation of the flavonoid biosynthetic pathway. PAL (L-phenylalanine ammonia-lyase); C4H (cinnamate 4-hydroxylase); 4CL (4-coumarate: Coenzyme-A ligase); CHS (chalcone synthase); CHI (chalcone isomerase); F3H (flavanone 3-hydroxylase); F3′H (flavanone 3’-hydroxylase); FS (flavone synthase); FLS (flavonol synthase); UGT (UDP-glycosyltransferase). The genes encoding enzymes in gray background have been identified in *G. biloba* in previous studies.

Although flavonoids in *G. biloba* have been utilized and investigated for centuries, their biosynthetic pathway has been poorly studied. Up to now, only a few structural genes in the upstream pathway have been identified by our or other groups, including *CHS* (*chalcone synthase*) (Pang et al., 2005), *CHI* (*chalcone isomerase*) (Cheng et al., 2011), *F3H* (*flavanone 3-hydroxylase*) (Shen et al., 2006a), and *ANR* (*anthocyanidin reductase*) (Shen et al., 2006b) (**Figure 1**). However, no *UGT* (*UDP-glycosyltransferase*) gene for the biosynthesis of flavonoid glucosides, the major flavonoid compounds in *G. biloba*, has been functionally characterized in this plant species.

UDP-glycosyltransferases belong to the large glycosyltransferases 1 family in the classification scheme that currently includes 98 groups (CAZy database^1^). UGTs have the conserved Plant Secondary Product Glycosyltransferase (PSPG) motif, a 44-amino-acid polypeptide, which is involved in the binding of UDP moiety of the sugar molecule (Gachon et al., 2005). Glycosylation catalyzed by UGTs affects the toxicity, stability, complexity, spectral characteristics and solubility of flavonoids (Vogt and Jones, 2000), and is often essential for flavonoid transport, storage and signal transduction (Jones and Vogt, 2001).

So far, extensive analyses of *UGT* genes have been carried out in several model and crop plants, such as *Arabidopsis*, maize, chickpea, *Lotus japonicus*, and tea (Bowles, 2002; Yonekura-Sakakibara and Hanada, 2011; Li et al., 2014; Sharma et al., 2014; Cui et al., 2016; Yin et al., 2017). However, considering the large number of *UGT* genes present in the plant genomes, the number of functional characterized *UGT* genes is still relatively small (Caputi et al., 2012). Furthermore, the evolutionary relationships among UGTs from different plant species and functional differentiation/diversification of UGT proteins remain unclear. A comprehensive genome-wide analysis of UGTs showed that expansion of UGTs occurred in both number and function during evolution in the plant kingdom (Yonekura-Sakakibara and Hanada, 2011). However, functional differentiation of UGTs in the same orthologous groups in the plant kingdom is still unclear (Yonekura-Sakakibara and Hanada, 2011; Caputi et al., 2012), and comprehensive information on UGTs from gymnosperms is still lacking. Therefore, as one of the four extant gymnosperm lineages (cycads, ginkgo, conifers, and gnetophytes) and a rich source of glycosylated flavonoids, *G. biloba* is an ideal plant for the investigation of functional diversification and differentiation of plant UGTs.

In the present study, we identified 25 *UFGT* (UDP:flavonoid glucosyltransferase) unigenes from a *G. biloba* leaf transcriptome and tested the *in vitro* function of eight full-length *UFGT* genes. In particular, recombinant UGT716A1 protein expressed in *E. coli* showed broad *in vitro* substrate specificity toward a wide range of flavonoid aglycones, including flavanol gallates and (methyl) gallic acid. Expression level of *UGT716A1* correlated with accumulation level of total flavonoids in different tissues of *G. biloba*. Sequence and enzymatic activity analyses of UGT716A1 homologs in *P. patens, A. thaliana, M. truncatula*, and *V. vinifera* revealed that ancestral plants like *P. patens* and *G. biloba* may have broader flavonoid substrate spectra than more advanced higher plants, suggesting that *UGT* genes experienced sub-functionalization and neo-functionalization during the expansion of the plant *UGT* superfamily.

## Results

### Analysis of *G. biloba* Leaf Transcriptome

To characterize genes involved in flavonoid biosynthesis in *G. biloba*, in particular *UGT* genes, we performed transcriptome sequencing with leaf tissue (deposited under BioProject ID: PRJNA353881 at NCBI). In total, 18,645,890 reads were obtained and 18,110,019 high-quality clean reads (97.13% of the raw data) remained after removal of the adaptor sequences, duplicate sequences, ambiguous reads, and low-quality reads. These reads were assembled into contigs ranging from 201 nt to 17,574 nt with an average length of 826 nt (Supplementary Figure S1A). Sequence data were aligned to the public protein databases (KO, KOG, and GO) using the BLASTX algorithm. Data were classified based on the putative proteins and a total of 24,593 sequences were annotated when E-value < e-5 (Supplementary Figure S1B). Among the 14 functional groups identified by KOG classification, secondary metabolites biosynthesis, transport, and catabolism counted for 3.8% (Supplementary Figure S1B).

To identify flavonoid biosynthetic pathway genes in *G. biloba*, the BLASTX results were searched for genes encoding enzymes involved in flavonoid biosynthesis. The unigenes related to this pathway encoded UFGTs (25 unigenes), F3H (13 unigenes), and FLS (flavonol synthase, 11 unigenes). In addition, several unigenes encoding C4H (cinnamate 4-hydroxylase), 4CL (4-coumarate: coenzyme-A ligase), CHS and CHI in the upstream pathway were also represented in the *G. biloba* leaf transcriptome (Supplementary Table S1).

### Sequence Analysis and Cloning of *UGT* Genes from *G. biloba*

In total, 121 unigenes annotated as glycosyltransferases or glucosyltransferase were identified in the *G. biloba* leaf transcriptome (Supplementary Table S2). Among them, 25 putative *UGT* unigenes ranging from 204 to 2,091 nt in length were annotated as flavonoid:UDP glucosyltransferases (Supplementary Table S3). Only one of them (comp25088_c0_seq1_13, designated as *UGT716A1*) represented a full-length gene in the transcriptome database. For comparative characterization purposes, we obtained the full-length sequences of another nine *UGT* genes*-UGT715A1* (comp14934_c0_seq1_3), *UGT717A1* (comp 310134_c0_seq1_2), *UGT721B1* (comp23937_c0_seq1_4), *UGT725A1* (comp24903_c0_seq1_38 and comp263434_c0_seq1_1), *UGT725B1* (comp38122_c0_seq1_15), *UGT726A1* (comp34006_c0_seq1_11), *UGT727A1* (comp103445_c0_seq1_19 and comp143607_c0_seq1_2), *UGT73AS1* (comp215683_c0_seq1_16), *UGT92K1* (comp37969_c1_seq1_10), using the available *G. biloba* EST sequence information deposited in the Medicinal Plant Genomics Resource^2^ during 2013. Because the full-length of the other 15 *UGT* genes were not available at the time of analysis, and they were not investigated further in the present study. The lengths of the ORFs and deduced proteins of the 25 *GbUGTs* are listed in Supplementary Table S3.

**FIGURE 2:**
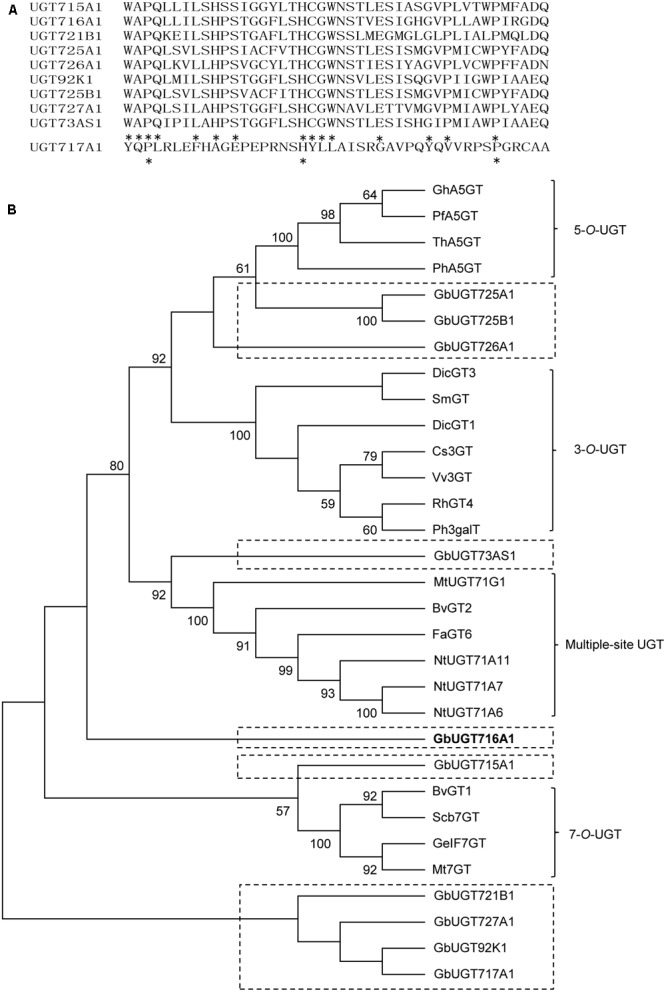
Sequence and phylogenetic analyses of the 10 deduced GbUGT proteins. **(A)** Multiple sequence alignments of the PSPG boxes of the 10 deduced GbUGT proteins. Asterisks indicate identical amino acids. **(B)** Phylogenetic analyses of the 10 deduced GbUGT proteins with functionally known UGTs from other plant species. Protein sequences were aligned with Clustal W and a neighbor-joining tree was constructed by using MEGA 6.0. Scale bar indicates the number of amino acid substitutions.

The 10 full-length deduced GbUGT proteins showed around 26–61% identity between each other at the amino acid level (Supplementary Table S4), 25–36% identity to UGT71A6 from tobacco at the amino acid level, 26–39% identity to UGT72L1 and UGT71G from *M. truncatula*, and 27–36% identity to UGT73B3 from *A. thaliana* (Supplementary Figure S2). Except for UGT717A1, the other nine deduced GbUGT proteins shared twelve identical amino acids within the conserved PSPG motif, but only three identical amino acids if UGT717A1 was included (**Figure 2A**), which might be a pseudogene. Eight of the 10 deduced GbUGT proteins shared the last glutamine (Q) residue within the PSPG motif that is believed to confer specificity for UDP-glucose as sugar donor (Kubo et al., 2004).

Phylogenetic analysis showed that the 10 GbUGT proteins were grouped into different clades comprising UGTs that display activity specific toward flavonoid 3-OH, 5-OH, 7-OH, or multiple OH positions (**Figure 2B**). Notably, GbUGT proteins were separated from other UGTs in each clade (**Figure 2B**), consistent with their gymnosperm origin, indicating that these *G. biloba* UGTs were phylogenetically distinct from other UGT proteins characterized from angiosperms.

### *In Vitro* Functional Characterization of Recombinant UGTs from *G. biloba*

To determine the enzymatic activities of the 10 recombinant GbUGT proteins, their open reading frames (ORFs) were amplified with corresponding gene-specific primers (Supplementary Table S5) and cDNA prepared from leaves. Eight of them were successfully obtained by RT-PCR, except for UGT725B1 and UGT73AS1 that might be expressed at very low level in leaves. The ORFs of the eight GbUGTs were cloned into pMAL-C2X vector and expressed in *E. coli* strain Novablue as soluble proteins. The 8 purified recombinant GbUGT proteins (Supplementary Figures S3A,B) were tested *in vitro* with UDP-glucose as sugar donor, and 19 flavonoid aglycones as potential substrates (Supplementary Table S6 and Supplementary Figure S4).

**FIGURE 3:**
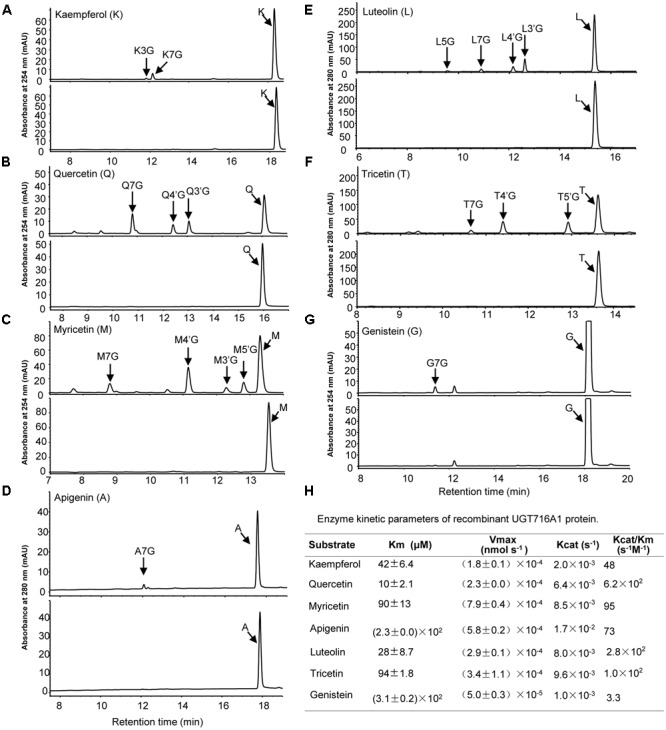
Analyses of the enzymatic products and kinetic parameters of recombinant UGT716A1 with flavonols, flavones, and isoflavones as substrates. **(A–G)** HPLC chromatograms of enzymatic products from assays of recombinant UGT716A1 (upper panels) and control (lower panels) with different substrates. **(A)**, kaempferol; **(B)**, quercetin; **(C)**, myricetin; **(D)**, apigenin; **(E)**, luteolin; **(F)**, tricetin; **(G)**, genistein. Arrowheads indicate the enzymatic products with corresponding substrates. K3G, K7G, Q7G, A7G, L7G, G7G were determined with authentic standards, and the others were predicted with mass spectrum, retention time and references. **(H)** The kinetic parameter of UGT716A1 with flavonols, flavones, and isoflavones as substrates. Values show the means from triplicate analytical replicates.

Recombinant UGT716A1 protein displayed a broad range of activities toward flavonols (kaempferol, quercetin myricetin), flavones (apigenin, luteolin and tricetin), and isoflavonoids (genistein), whereas recombinant UGT92K1 protein only displayed activity toward genistein (Supplementary Table S6). All the remaining recombinant GbUGT proteins did not exhibit activity toward any of the tested flavonoid aglycones (Supplementary Table S6). Multiple peaks appeared on HPLC in the reactions with recombinant UGT716A1 protein and all substrates except apigenin and genistein (**Figures 3A–G**, upper panels), whereas no product peak was observed from control reactions without recombinant UGT716A1 protein (**Figures 3A–G**, lower panels). The enzymatic products were further analyzed by UPLC/MS, revealing that these enzymatic products all ostensibly lost one glucose moiety (m/z 162) to yield the corresponding aglycone (Supplementary Figures S5A–R). This indicates that the enzymatic products are flavonoid mono-glucosides that are glucosylated on different OH-groups (**Figures 3D,G**). The enzymatic reaction product with genistein as acceptor for recombinant UGT92K1 was identified as genistein 7-*O*-glucoside on comparison to an authentic reference standard (Supplementary Figure S6).

### Enzymatic Properties of Recombinant GbUGT716A1 Protein

GT716A1 protein exhibited different kinetic parameters toward flavonols, flavones, and isoflavones, with relatively low *K*_m_ values for quercetin and luteolin (10 and 28 μM, **Figure 3H**), but relatively weak affinity with higher *K*_m_ values of 230 and 310 μM for apigenin and genistein, respectively (**Figure 3H**). Thus, UGT716A1 has a substrate preference, although it can utilize multiple flavonoid substrates.

**FIGURE 4:**
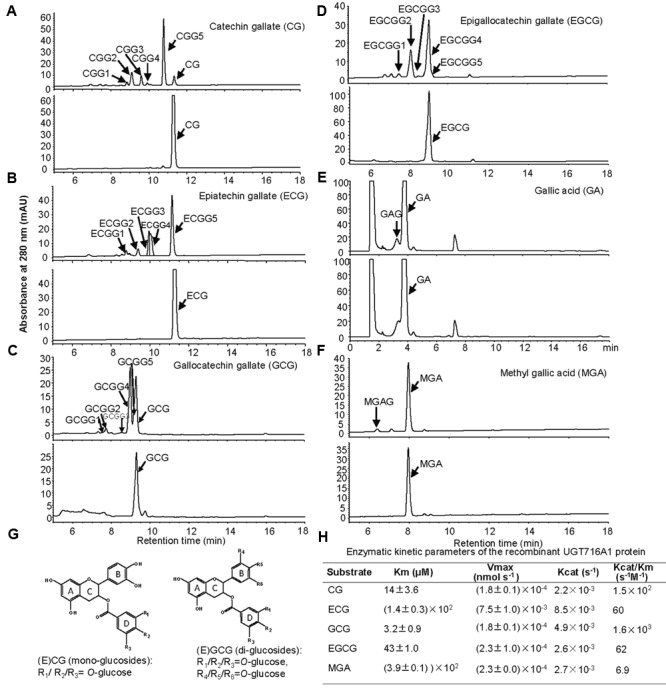
Analyses of the enzymatic products and the enzymatic kinetic parameter of the recombinant UGT716A1 protein with flavanol gallates and gallic acids as substrates. **(A–F)** HPLC chromatograms of enzymatic products from enzymatic assays of recombinant UGT716A1 protein (upper panels) and control (lower panels) with various substrates. **(A)**, catechin gallate; **(B)**, epicatechin gallate; **(C)**, gallocatechin gallate; **(D)**, epigallocatechin gallate; **(E)**, gallic acid; **(F)**, methyl gallic acid. Arrowheads indicate the enzymatic products with the corresponding substrates at a concentration of 100 μM. **(G)** Proposed schematic structures of glucosides of flavanol gallates. **(H)** The kinetic parameter of UGT716A1 protein with flavanol gallates and (methyl) gallic acids as substrates and UDP-glucose as sugar donor. Values show the means of analytical triplicates.

Notably, recombinant UGT716A1 protein showed activity toward flavanol gallates (**Figures 4A–D**). HPLC analysis showed that multiple product peaks were observed with catechin gallate (CG), epicatechin gallate (ECG), gallocatechin gallate (GCG), and epigallocatechin gallate (EGCG) (**Figure 4**). However, no product was observed with non-galloylated flavanols as substrates (catechin, gallocatechin, epicatechin, and epigallocatechin, **Table 1**). Mass spectra generated by UPLC/MS analysis confirmed that both CG and ECG can be glycosylated on different OH groups to produce mono-glucosides that yielded a molecular ion at m/z 603 (Supplementary Figures S7A–J). GCG and EGCG could be glycosylated at two OH groups to produce five di-glucosides, which yielded molecular ions of m/z 619 and 781 (Supplementary Figures S8A–J).

**Table 1:** Activities of several recombinant UGT proteins toward various classes of flavonoid and gallic acid substrates.

Class	Substrate	PpUGT1	PpUGT2	PpUGT3	UGT716A1	VvUGT1	VvUGT3	MtUGT1	MtUGT2
Flavonols	Kaempferol	(58.5 ± 5.9)%	ND	(85.0 ± 7.6)%	(16.3 ± 1.3)%	(43.9 ± 10.6)%	ND	ND	ND
	Quercetin	(48.4 ± 2.9)%	ND	(61.0 ± 3.8)%	(57.5 ± 0.8)%	(54.8 ± 4.1)%	(1.6 ± 0.4)%	ND	ND
	Myricetin	(6.6 ± 0.3)%	ND	(40.3 ± 6.4)%	(54.1 ± 2.8)%	(54.1 ± 2.6)%	ND	ND	ND
Flavones	Apigenin	(4.9 ± 0.2)%	ND	ND	(1.9 ± 0.2)%	ND	ND	ND	ND
	Luteolin	(9.9 ± 0.6)%	ND	ND	(47.1 ± 2.0)%	ND	ND	ND	ND
	Tricetin	(9.8 ± 0.7)%	ND	ND	(20.7 ± 2.5)%	ND	ND	ND	ND
Isoflavones	Daidzein	ND	Trace	ND	ND	ND	ND	ND	ND
	Genistein	(6.2 ± 0.3)%	(1.1 ± 0.0)%	ND	(1.1 ± 0.3)%	ND	ND	ND	ND
Flavanols	Catechin	(89.1 ± 2.0)%	ND	ND	ND	ND	ND	ND	ND
	Epicatechin	(86.5 ± 0.4)%	ND	ND	ND	ND	ND	ND	ND
	Gallocatechin	ND	ND	ND	ND	ND	ND	ND	ND
	Epigallocatechin	ND	ND	ND	ND	ND	ND	ND	ND
Flavanol gallates	Catechin gallate	ND	ND	ND	(36.1 ± 10.0)%	ND	Trace	ND	ND
	Gallocatechin gallate	ND	ND	ND	(4.5 ± 1.3)%	ND	Trace	ND	ND
	Epicatechin gallate	Trace	ND	ND	(76.2 ± 1.0)%	(2.2 ± 0.1)%	Trace	ND	ND
	Epigallocatechin gallate	ND	ND	ND	(100 ± 0.0)%	ND	Trace	ND	ND
Gallic acid	Methyl gallic acid	(99.2 ± 0.3)%	ND	ND	(4.7 ± 0.1)%	(99.3 ± 0.9)%	ND	ND	ND

*ND, not detected. Percentage represents the means of substrate converted from enzymatic assays at the concentration of 100 μM of three independent replications. “Trace” means the conversion rate was below 1%.*

No commercial flavanol gallate glucoside standards are available, and the reaction products were very close on HPLC even a number of conditions were tested, making further purification difficult. Therefore, we compared the enzymatic product EGCGG5 with EGCG-4′,4″-O-β-D-gluco-pyranoside (EGCG-4′,4″-Glu) that was chemically synthesized and provided by Wang group (Zhang et al., 2016). Authentic EGCG-4′,4″-Glu co-eluted with EGCGG5 on HPLC (Supplementary Figure S9A), and showed an identical UV spectrum to EGCGG5 along with EGCGG1, 2, 3, and 4 (Supplementary Figure S9). Together, our results indicate that one of the enzymatic products of UGT716A1 is EGCG-4′,4″-Glu, and that the others are di-glucosides that are glucosylated at different OH-positions.

It is also possible that one or more OH groups of the gallic acid moiety of EGCG could be glycosylated. In order to further test this possibility, gallic acid was tested as potential substrate, and a new peak (not the carboxylic ester β-glucogallin) eluted prior to gallic acid on HPLC (**Figure 4E**). Because gallic acid is highly hydrophilic, it eluted very early on HPLC, and the enzymatic product is very close to gallic acid, making separation difficult (**Figure 4E**). Therefore, methyl gallic acid that is less hydrophilic than gallic acid was also tested as a potential substrate, and a new product peak was detected on HPLC as compared to the control (**Figure 4F**). Mass spectra of the glycosylation products with gallic acid and methyl gallate as substrates had molecular ions at m/z 331 and 345, respectively, implying single glycosylation at the OH groups of C3, C4, or C5 on gallic acid (**Figure 4G** and Supplementary Figures S10A,B). Taken together, these results indicate that the glycosylation position for CG and ECG is most likely occur at one of the three OH-groups (R1/R2/R3) on the D ring (**Figure 4G**, left), while the di-glycosylation positions for GCG and EGCG are most likely one of the three OH groups on the B ring (R_4_/R_5_/R_6_) and a second one on the D ring (R_1_/R_2_/R_3_, **Figure 4G**, right).

Enzyme kinetic analysis showed that UGT716A1 had the highest affinity to GCG, with a *K*_m_ value of 3.2 μM and *K*_cat_*/K*_m_ value of 1.6 × 10^3^ s^-1^M^-1^, followed by CG and EGCG with *K*_m_ values of 14 and 43 μM, and *K*_cat_*/K*_m_ values of 1.5 × 10^2^ s^-1^M^-1^ and 62 s^-1^M^-1^, respectively (**Figure 4H**). Methyl gallic acid showed the weakest affinity with the highest *K*_m_ value of 390 μM and *K*_cat_*/K*_m_ value of 6.9 s^-1^M^-1^, respectively (**Figure 4H**). These results indicate that recombinant UGT716A1 protein shows strong preference for flavanol gallates as substrates.

Because flavonoids present in *G. biloba* are mainly glucosides, with very few of them are galactosides or rhamnosides (Liu et al., 2015), we then detect the activity of UGT716A1 with commercial available UDP-galactose as sugar donor. It showed that UGT716A1 protein exhibited activity toward flavonols, flavones, flavanol gallates, and MGA, but the conversion rate were relatively lower than those with UDP-glucose as sugar donor (Supplementary Table S7), indicating UGT716A1 prefer UDP-glucose as sugar donor than UDP-galactose.

### Temporal and Spatial Expression of *UGT716A1* Transcripts

**FIGURE 5:**
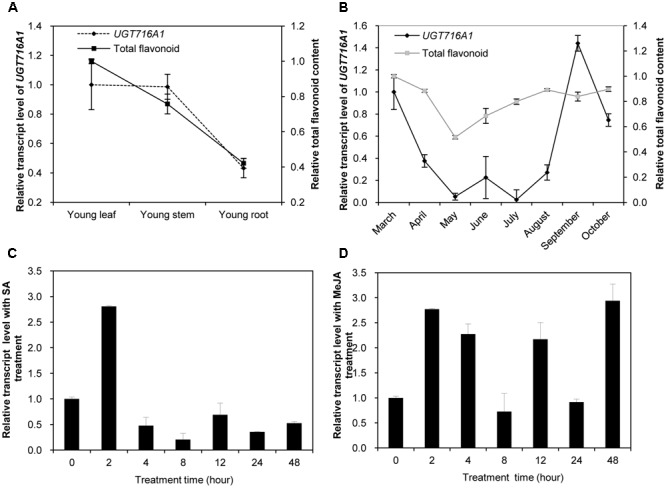
Relative transcript levels of *UGT716A1* and flavonoid content in *G. biloba*. **(A,B)** Relative transcript levels of *UGT716A1* and flavonoid content in various tissues **(A)** and leaves at different seasons **(B)** of *G. biloba*. The total flavonoids were relatively quantified with standard curve of quercetin, and 34.8 μg quercetin/mg dry weight in leaves of April was set as value of 1 in **B**. **(C,D)** Relative transcript level of *UGT716A1* after SA **(C)** and MeJA **(D)** treatment of *G. biloba* suspension cells. Transcripts were detected by qRT-PCR. Values show the means and standard deviations of analytical triplicates.

Because *UGT716A1* encodes an enzyme with multiple substrates, further assessments of its potential *in vivo* function was made by determining its transcript in roots, stems, and leaves from young seedlings, as well as leaves from the adult tree. The transcript levels of *UGT716A1*, as determined by quantitative real-time PCR (qRT-PCR), were similar in young leaves and stems, where they were slightly higher than in roots of young seedlings (**Figure 5A**). The relative transcript level of *UGT716A1* in leaves of adult tree peaked during September but decreased again during October with low expression between May and July (**Figure 5B**). Total flavonoid contents were higher in young leaves and stems than in roots (**Figure 5A**), and increased steadily from May to October (**Figure 5B**). The relative transcript level of *UGT716A1* showed a significant positive correlation with total flavonoid content from April to September (*R* = 0.546, *p* < 0.05, by Pearson correlation analysis, **Figure 5B**), although the changes in flavonoids did not mirror the dip to virtually zero level of *UGT716A1* transcripts in July. Therefore, UGT716A1 could possibly be a major contributor to the accumulation of flavonoid glucosides in *G. biloba* leaves.

Salicylic acid (SA) and methyl jasmonate (MeJA) are key plant hormones that play crucial roles in inducible defenses against microbial pathogens and insect herbivores in plants (Pieterse et al., 2009; Leon-Reyes et al., 2010; Verhage et al., 2010). Several flavonoids and corresponding pathway genes are known to be induced by SA and/or MeJA (Griesser et al., 2008; Xu et al., 2009; Yin et al., 2017). To determine whether *UGT716A1* was also inducible by SA or MeJA, its transcript levels were measured in *G. biloba* suspension cells exposed to these two hormones. *UGT716A1* transcripts increased by more than two-fold after SA treatment for 2 h (**Figure 5C**). But the response to MeJA was more complex, with similar fold changes but suggestion of an oscillating response up 48 h post-treatment (**Figure 5D**). The increased expression of *UGT716A1* in response to SA and MeJA treatments suggested that *UGT716A1* may be involved in biotic defense in *G. biloba*.

### Over-Expression of *UGT716A1* in *A. thaliana*

**FIGURE 6:**
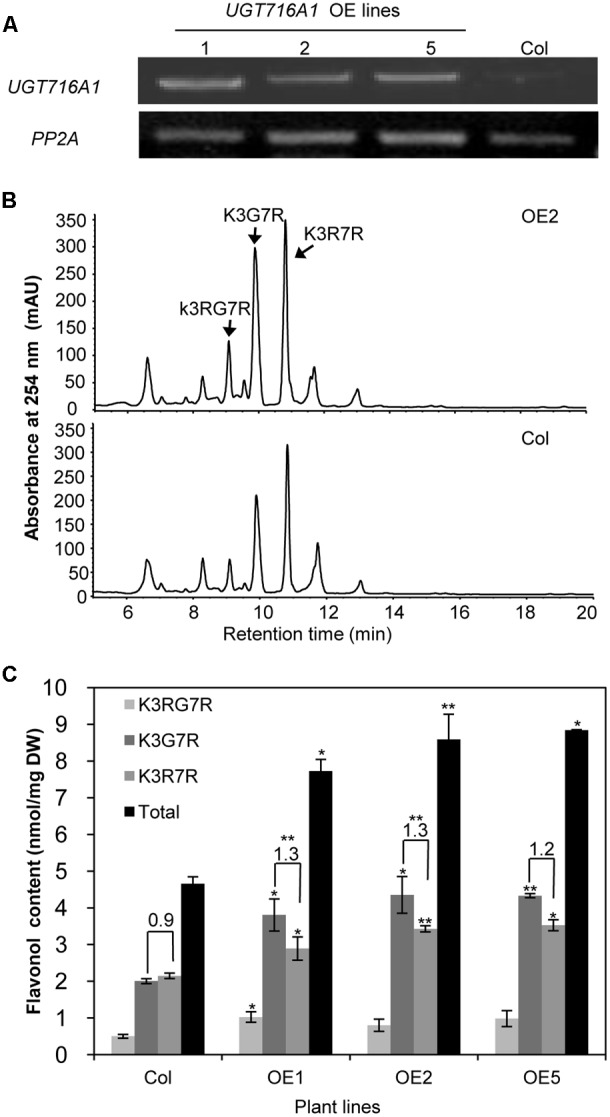
Ectopic expression of *UGT716A1* in *A. thaliana*. **(A)** Transcript levels of *UGT716A1* in seedlings of independent *A. thaliana* transgenic lines detected by RT-PCR with the house-keeping gene *PP2A* as control; **(B)** HPLC chromatograms of flavonoid profiles in transgenic line (OE2) and wild type control line in 10-day-old seedlings; **(C)** Flavonol contents in 10-day-old seedlings of the transgenic lines and wild type control. Values show the means and standard deviations of triplicate analytical replicates from transgenic lines and control. Asterisks indicate that a value is significantly different from that of the control line by Student’s *t*-test (^∗^*P* < 0.05; ^∗∗^*P* < 0.01).

To test how broad the flavonoid substrate specificity of UGT716A1 may be *in vivo*, it was ectopically expressed in *A. thaliana*, driven by the 35S promoter. A high expression level of *UGT716A1* in three independent homozygous lines (OE1, OE2, and OE5) was confirmed by qRT-PCR and these lines were selected for further analysis (**Figure 6A**).

Kaempferol-3-*O*-[rhamnosyl (1→2glucoside)]-7-*O*-rhamnoside (K3RG7R), kaempferol-3-*O*-glucoside-7-*O*-rhamnoside (K3G7R), and kaempferol-3-*O*-rhamnoside-7-*O*-rhamnoside (K3R7R) were the three major flavonol glycosides detected in 10-day-old *A. thaliana* seedlings (**Figure 6B** and Supplementary Figure S11). Among them, levels of K3G7R and K3R7R increased in all three transgenic lines compared with the wild type control (**Figure 6B**). Contents of K3G7R, K3R7R, and total flavonol glucosides increased by 1.9–2.2, 1.3–1.6, and 1.7–1.9 fold, in transgenic lines as compared to the wild type control (**Figure 6C**). The ratio of K3G7R to K3R7R content increased in transgenic lines (1.3-, 1.3-, and 1.2-fold in OE1, OE2, and OE5) compared with the wild type control (0.9, **Figure 6C**), indicating that the flux to kaempferol rhamnosides was switched to formation of glucosides by over-expression of *UTG716A1*. In seeds, the flavonoid profiles, total flavonoid content and relative proanthocyanidin content did not change significantly in transgenic lines compared with the wild type control (Supplementary Figure S12).

### Identification and Characterization of *UGT716A1* Homologous Genes

To further explore the functional evolution of *UFGT* genes, we analyze 1, 21, and 142 *UGT* genes from the primitive plants *C. reinhardtii, P. patens*, and *S. moellendorffii*, respectively (Yonekura-Sakakibara and Hanada, 2011), and identified the homologs to UGT716A1 from *C. reinhardtii* (*CreUGT*), *P. patens* (*PpUGT1, 2, 3*), *S. moellendorffii* (*SmUGT1, 2, 3*). Because *UGTs* among different plant species showed low identity, only the homologs with the best blastp matches were selected from these plant species. We also identified UGT716A1 homologs from model plant species with available genome sequences, including three *UGTs* from *A. thaliana* (*AtUGT73B3, B4, B5*), three from *M. truncatula* (*MtUGT1, 2* and UGT72L1) and three from *V. vinifera* (*VvUGT1, 2, 3*). A phylogenetic tree with these UGTs showed that CreUGT branched earlier, followed by a cluster comprising UGT716A1, MtUGT1, and MtUGT2 (Supplementary Figure S13). Most *UGT* genes do not contain introns, however, analysis of gene structures showed that lower plants such as *C. reinhardtii* (eight introns) and *P. patens* (four introns for *PpUGT2* and three introns for *PpUGT3*) have more introns than the others (Supplementary Figure S13B). In order to verify the genome sequence of UGT716A1, it was also amplified with genome DNA. Comparison of genome and cDNA sequence indicated that UGT716A1 does not have any intron. By contrasting with *C. reinhardtii* and *P. patens*, higher plant UGT *MtUGT1, AtUGT73B3*, and *VvUGT1*, like *UGT716A1*, do not have any introns (Supplementary Figure S13B), characteristic of UGT71 family members (Paquette et al., 2003).

In order to address the substrate specificity of these *UGT*716A1 homologs, we isolated and expressed PpUGT1, PpUGT2, PpUGT3, MtUGT1, MtUGT2, VvUGT1, and VvUGT3 in *E. coli* (Supplementary Figure S3C). The activity of these seven recombinant proteins was then tested with UDP-glucose as sugar donor and various flavonoid aglycones as potential acceptor substrates. Recombinant PpUGT1 protein from *P. patens* had a broader substrate spectrum than UGT716A1, displaying activities toward at least six classes of flavonoid substrates, including flavonols (kaempferol, quercetin, and myricetin), flavones (apigein, luteolin, and tricetin), isoflavones (genistein), (epi)-catechins (catechin and epicatechin), ECGs, as well as methyl gallic acid (Supplementary Figures S14A–L, S15, and **Table 1**). In contrast, the recombinant PpUGT2 and PpUGT3 proteins had more restricted specificity being active toward isoflavones (genistein and daidzein) and flavonols (kaempferol, quercetin, and myricetin), respectively (Supplementary Figures S14M–Q and **Table 1**). To explore possible presence of flavonoid glucosides in *P. patens*, we analyzed the flavonoid compounds in *P. patens* using UPLC/MS, and detected a few putative flavonoid compounds, including kaempferol-hexoside and tricetin-rhamnoside (Supplementary Figure S16). This result indicates PpUGTs are most likely responsible for the biosynthesis of these flavonoid glycosides in *P. patens.*

**FIGURE 7:**
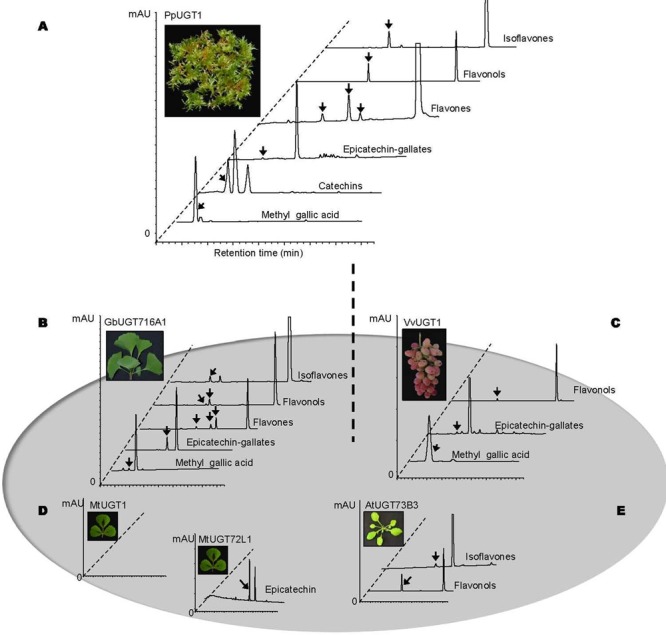
Summary of enzymatic activities of recombinant UGT proteins from several model plants. **(A–E)** Representative HPLC chromatograms of enzymatic assays with recombinant UGT proteins and various classes of substrates. **(A)** PpUGT1; **(B)** UGT716A1; **(C)** VvUGT1; **(D)** MtUGT1 and MtUGT72L1; **(E)** AtUGT73B3. All the representative HPLC chromatograms were from authentic *in vitro* assays in the present study except for AtUGT73B3 that were adapted from previous studies (Lim et al., 2004 and Weis et al., 2006).

Recombinant VvUGT1 protein from higher plant *V. vinifera* exhibited activities toward flavonols (kaempferol, quercetin, and myricetin), ECG and methyl gallic acid (Supplementary Figures S14R–V and **Table 1**), and VvUGT3 was active toward quercetin, and all the four epi-(gallo)-catechin gallates (Supplementary Figures S14W–AA and **Table 1**). However, MtUGT1 and MtUGT2, two proteins with highest identity with UGT716A1 in *M. truncatula*, showed no activity toward any of these flavonoids. Similar as reported in previous studies, UGT73B3, UGT73B4, UGT73B5 from *A. thaliana* exhibit activity toward quercetin and daidzein (Lim et al., 2004; Weis et al., 2006). In each case, these UGTs from higher plants appeared to have a narrow flavonoid substrate spectrum (**Figure 7**). Taken together, our data suggest that lower plants like *P. patens* have *UGT* genes encoding enzymes with broad substrates than those from higher plants, and UGTs with specific activity toward flavanol gallates occurred in lower plants like *P. patens.*

## Discussion

### Functional Significance of UGT716A1 in Glycosylated Flavonoid Diversity

In *G. biloba*, the majority of flavonoids are present in glycosylated forms (Liu et al., 2015), indicating the importance of *UGTs* in flavonoid metabolism of this species. Here, we revealed that UGT716A1 in *G. biloba* is capable of biosynthesizing a broad range of flavonoid glycosides, including glycosides of flavonols, flavones, isoflavones, and flavanols (**Table 1** and **Figures 3, 4**). Although hundreds of *UGT* genes have been identified in plants (Wang, 2009; Yonekura-Sakakibara and Hanada, 2011; Cui et al., 2016; Yin et al., 2017), few displayed broad substrate spectrum. For example, five UGTs from *M. truncatula* (GT22D, GT22E09, GT29C, GT29H, and GT83F) displayed activities toward at most three classes of flavonoids, including flavonols, flavones, and isoflavones (Modolo et al., 2007); UGT72L1 is an epicatechin-specific UGT (Pang et al., 2008), which is different from the other five UGTs in *M. truncatula*. UFGT with probably the broadest flavonoid substrate spectrum from *A. thaliana*, UGT73C6, can use three classes of flavonoid aglycones as substrates, including flavonols (kaempferol and quercetin), a flavone (apigenin), and an isoflavone (genistein) (Jones et al., 2003). *In vitro* assays revealed that UGT716A1 is a multi-substrate UFGT with broad *in vitro* flavonoid substrate spectrum.

Several flavonol and flavone glycosides, including kaempferol 3-*O*-glucoside, kaempferol 7-*O*-glucoside, apigenin 7-*O*-glucoside and luteolin 3′-*O*-glucoside, were detected in *G. biloba* (Victoire et al., 1988; Singh et al., 2008; Liu et al., 2015), all consistent with the *in vitro* activities of recombinant UGT716A1 protein. Although catechin, epicatechin, gallocatechin, and epigallocatechin aglycones have been detected in *G. biloba* (Liu et al., 2015), their gallates and glucosides were not reported. Their levels may be below detection limits, or they may have been missed because they were unexpected. Because of the seemingly unique ability of UGT716A1 to glycosylate these compounds, detailed tissue-specific metabolomic analysis of *G. biloba* is warranted. It is also possible that the *in vivo* activity of UGT716A1 does not match its *in vitro* activity, a situation that has been reported for many plant UGT proteins, including UGT73C6 from *A. thaliana*, UGT78G1 from *M. truncatula* and UGTs from *L. japonicus* (Jones and Vogt, 2001; Modolo et al., 2007; Peel et al., 2009; Husar et al., 2011; Yin et al., 2017). It is the same reason that when *UGT716A1* was over-expressed in *A. thaliana* (**Figure 6**), no extensive and obvious flavonoid glucosides were detected except for the contents of K3G7R and K3R7R.

The expression profile of *UGT716A1* corresponded with the accumulation pattern of total flavonoids in different tissues, and partially corresponded with flavonoid levels in leaves during different seasons (**Figures 5A,B**). Therefore, *UGT716A1* possibly encodes a major *UGT* for the accumulation of flavonoid glycosides in *G. biloba*, although this can only be determined unequivocally by wide genetic analysis. In contrast, the other seven GbUGTs without any activity toward these tested flavonoids may be functionally inactive genes in the expansion and evolution of *UFGTs* in *G. biloba*.

The transcript level of *UGT716A1* was also inducible by SA and MeJA treatments (**Figures 5C,D**), similar to its homologs AtUGT73B3 and AtUGT73B5, AtUGT73B3 and AtUGT73B5 play important roles in the hypersensitive responses of *Arabidopsis* to bacterial pathogens (Langlois-Meurinne et al., 2005; Simon et al., 2014). The similar expression pattern and close relationship of *UGT716A1* with *AtUGT73B3* and *AtUGT73B5* implies that *UGT716A1* may function in defense responses in *G. biloba*, the mechanism of which requires further investigation.

Glycosylated flavonoids are multifunctional polyphenolic compounds that play important roles in plant defense and are found in essentially all higher plant species (Yonekura-Sakakibara and Hanada, 2011), and their production is one of the three major denfense systems in *G. biloba* for its response to herbivore attack (Guan et al., 2016). Although a significant number of putative *UGT* genes have been identified in the *G. biloba* genome, the numbers of functionally characterized *UGT* genes is still rather small. Interrogation of the now available genome sequence of *G. biloba* will enrich our understanding of flavonoid evolution in the plant kingdom.

### Significance of UGT716A1 in the Functional Diversification and Differentiation of UGTs in Plants

The expansion of genes in the plant kingdom has been attributed to duplication events that have occurred during the evolution of land plants (Lynch and Conery, 2000; Lockton and Gaut, 2005; Hanada et al., 2008). The expansion of *UGT* genes is believed to have occurred rapidly after the divergence of bryophyte *P. patens*, the genome of which possess at least 12 putative *UGT* genes as compared to 1 from *C. reinhardtii* (Yonekura-Sakakibara and Hanada, 2011).

In lower plants like *P. patens*, even only few putative flavonoids were detected, but these plants still keep the functional flavonoid pathway genes, like the recently identified type II CHI that was previously believed to be specific for legume plants (Cheng et al., 2017). Similarly, *P. patens* has more *UGTs* genes, and they constitutes approximately 0.03% of the total number of genes. In comparison, more than 60 flavonoids were detected *G. biloba* (Liu et al., 2015), and *UGT* counts for 0.46% of the total number of genes. For *A. thaliana, M. truncatula*, and *V. vinifera*, the numbers were 0.46%, 0.39%, and 0.54% (Caputi et al., 2012; Yin et al., 2017), with flavonoids of 54, 36, and 52, respectively (Staszków et al., 2011; Saito et al., 2013; De Rosso et al., 2015; Wei et al., 2017). It is clear that UGT family significantly expanded in higher plants, resulting in diverse flavonoid compounds, which is associated with their adaption during evolution.

In the present study, we also found that PpUGT1 from the bryophyte *P. patens* displays a broader substrate spectrum for flavonoid compounds than does UGT716A1, although flavonoid compounds were not extensively detected in *P. patens* in previous studies (Wolf et al., 2010; Kumar and Pandey, 2013). However, we detected presence of flavonoid glucosides in *P. patens* in the present study. *UGTs* for flavonoid glycosylation therefore evolved at a very early period of land plant colonization, and later evolved through gene duplication in higher plants.

Homology genes to *UGT716A1* in other higher plants, including *A. thaliana, M. truncatula*, and *V. vinifera*, encode UGTs that only show activities toward a few or none of the substrates of *UGT716A1*, suggestive of functional specification on *UGTs* from their ancestral genes (Lynch and Conery, 2000; Lockton and Gaut, 2005; Hanada et al., 2008). It is most likely that *UGTs* involved in flavonoid glycosylation have undergone sub-functionalization and neo-functionalization from their ancestral *UGT* gene, as with other plant genes (Moore and Purugganan, 2005).

*UGT716A1* does not have any introns, common with its top homologs in *A. thaliana (UGT72B3*), *M. truncatula (MtUGT1)* and *V. vinifera* (*VvUGT1*, Supplementary Figure S13), and this is the characteristic of UGT72 and 71 family (Paquette et al., 2003). But the UGTs from lower plants have more introns, like *P. patens* and *C. reinhardtii* (Supplementary Figure S13). This may due to the independent loss of intron duplication by retrotransposition in which mRNA from a parental gene can be inserted into chromosomal DNA as an intron-less form by a reverse transcriptase enzyme (Yonekura-Sakakibara and Hanada, 2011), which will require further investigation.

### Significance of UGT716A1 for the Bioengineering of Bioactive Flavonoid Glucosides

Flavanol gallates, especially EGCG, have shown significant bioactivity relating to human health (Yang et al., 2009; Yuan et al., 2011; Chowdhury et al., 2016). However, the use of these compounds, represented by EGCG, was often hindered by poor water solubility, rapid metabolism and ready degradation in aqueous solutions (Kitao et al., 1995; Hong et al., 2002; Zhang et al., 2016). In comparison, the glucosylated flavanol gallates exhibit similar antioxidant properties, yet have increased solubility in water and stronger tyrosinase inhibitory effects, suggesting that these forms may be superior to flavanol gallates aglycones for application as food additives, drugs, and cosmetics (Moon et al., 2006; Zhang et al., 2016). To date, glycosylation of flavan-3-ols gallates have often been carried out *via* chemical conversion or use of glycosyltransferases of bacterial origin (Kitao et al., 1995; Moon et al., 2006; Hyun et al., 2007; Zhang et al., 2016).

In the present study, we firstly discovered that recombinant UGT716A1 protein exhibits unique glycosylation activity toward flavanol gallates, including CG, ECG, GCG, and EGCG. UGT716A1 is distinct from UGT72L1 (identified in *M. truncatula*) that was previously shown to glucosylate only non-galloyated epicatechin (Pang et al., 2008). Furthermore, recombinant UGT716A1 is able to produce multiple glucosylated products with a single flavanol substrate, whereas UGT72L1 only produces a single glucosylated product (Pang et al., 2008). These feathers, plus the fact that recombinant UGT716A1 has a high affinity for flavanol substrates, suggesting that the enzyme could be used as an environmentally friendly and efficient biological catalyst for the production of flavanol gallate glucosides of plant origin. Although over-expression of *UGT716A1* did not successfully produce equal type of flavonoid glucosides in *A. thaliana*, it will be ideal to transform UGT716A1 into other plants with high flavanols level (e.g., tea or grape), for the production of bioactive flavonol glucosides. Nevertheless, the production of diverse bioactive flavan-3-ol gallate glucosides by UGT716A1 illustrates an economical biosynthetic strategy to create novel natural products with potential for use in food, drug, and cosmetics applications.

## Materials and Methods

### Plant Materials and Chemicals

Young leaves of a *G. biloba* tree growing in the Beijing Botanical Garden were collected during April, 2012, then immediately frozen and kept at -80°C for RNA extraction and transcriptome sequencing. Roots, stems, and leaves of young seedlings, and leaves from the same tree at different seasons in the year 2013 were collected for RNA extraction and flavonoid analysis.

All authentic substrates were purchased from Shanghai Tongtian Biotechnology Company (Shanghai, China). Maltose-binding resin for protein purification was purchased from New England Biolabs (Frankfurt, Germany). All solvents used for HPLC and UPLC/MS/MS were of analytical grade.

### RNA Extraction and Transcriptome Sequencing

Total RNAs from *G. biloba* tissues were extracted using the CTAB method (Liao et al., 2004), followed by digestion with DNase I (Ambion, United States) at 37°C for 1 h. mRNA from young leaves was extracted with Micropoly (A) Purist^TM^ mRNA purification kit (Ambion, United States) according to the manufacturers’ instruction. Ten micrograms of mRNA was used for library construction and subsequent transcriptome sequencing in Hanyu Biotechnology Co., Ltd. (Shanghai, China).

### RNA Sequencing, Assembly, and Annotation

In order to get clean reads for *de novo* assembly and further analyses, all raw reads from RNA-seq were assembled with Trinity (Grabherr et al., 2011). The EMBOSS toolbox was used to find the amino acid sequence of contigs (Rice et al., 2000). Those amino acid sequences were further used for blastp by comparison with GenBank Nr (NCBI non-redundant protein sequences), GO (Gene Ontology), KEGG (Kyoto Encyclopedia of Genes and Genomes), and KOG (euKaryotic Ortholog Groups)/COG (Clusters of Orthologous Groups) database, with E-value < 1e-5. The GO predictions were performed with the Swiss-Prot and TrEMBL database with blastp and E-value < 1e-5; the blastp results were then input to Gopipte according to the gene2go program to obtain the GO information for the top match predicted proteins. By key word search with “glycosyltransferase” or “glucosyltransferase” 121 GT unigenes were obtained. Among them, 25 were annotated as UDP:flavonoid glucosyltransferase.

### Sequence Alignment and Phylogenetic Analysis of *GbUGT* Genes

Multiple sequences alignments of target *GbUGTs* were performed using CLUSTAL W, and the phylogenetic trees were constructed using MEGA 6.0 (Tamura et al., 2013). The neighbor-joining statistical method was used to calculate the phylogenetic tree (Van de Peer and De Wachter, 1994), with 1,000 bootstrap replications. Distance calculation was performed with Poisson correction and branch lengths were shown only when values were above 50%.

### Cloning and Gene Expression Analysis

The ORF of *UGT* genes from *G. biloba, M. truncatula*, and *V. vinifera* were obtained by PCRs with cDNAs prepared from leaves, and those of *C. reinhardtii, P. patens*, and *S. moellendorffii* were amplified from cDNA prepared from whole plants (primers listed in Supplementary Table S5). The ORFs of these *UGT* genes were amplified using *pfx* high fidelity DNA polymerase in a total volume of 50 μL at 94°C for 3 min; 35 cycles of 94°C for 45 s, 52–60°C for 50 s, and 68°C for 90 s; followed by a final extension of 72°C for 10 min. The amplified ORFs with restriction sites were digested with the corresponding restriction enzymes, and ligated into expression vector pMAL-c2X (New England Biolabs, Germany). After confirmation for correct insertions by sequencing, the recombinant plasmids (pMAL-c2X-UGTs) were introduced into *E. coli* strain Novablue.

CDNAs prepared from various tissues of *G. biloba* were used for qRT-PCR. Quantitative RT-PCR analyses were carried out with triplicates using SYBR Green reagent (Kapa, United States) according to the manufacturer’s instructions. qRT-PCRs were performed with primers specific for *UGT716A1* (UGT716A1RTF and UGT716A1RTR) and produced single product with expected size. The qRT-PCR conditions were as described previously (Jiang et al., 2015). The *PP2A* house-keeping gene was used as an internal control for normalization, and the primers for *PP2A* gene were PP2A-F and PP2A-R.

### Expression, Purification, and Enzymatic Assay of Recombinant UGT Proteins

The Novablue strains harboring the pMAL-c2X-UGTs plasmids were cultured at 16°C, and the recombinant proteins induced with 0.3 mM isopropyl-β-D-thiogalactoside (IPTG) for 24 h and purified according to the pMAL Protein Fusion and Purification System (New England Biolabs, Germany). The purified proteins were further concentrated with molecular sieve (30 kDa, Millipore, United States). Protein concentration was determined as previously described (Bradford, 1976), and the presence of the recombinant proteins confirmed on 12% SDS-PAGE.

Enzymatic assays were carried out at 30°C for 30 min in a total volume of 50 μL containing 4 mM UDP-glucose (or UDP-galactose), 100 μM substrate, and 2–5 μg purified UGT proteins in Tris-HCl buffer (100 mM, pH7.0). The reactions were stopped by the addition of the same volume of methanol, and 40 μL mixtures were injected for HPLC analysis after centrifugation at 14,000 rpm for 5 min. The corresponding aglycones were used for quantification of conversion rate.

To determine enzyme kinetic parameters, substrates at concentrations of 0, 25, 50, 100, 200, and 400 μM were used in the aforementioned assay in triplicate. Enzymatic products were determinate by HPLC (Agilent 1260) as described previously (Jiang et al., 2015). Enzymatic products were further confirmed on UPLC/MS (Waters, United States) as previously described (Wu et al., 2016). All compounds were detected at a wavelength of 254 or 280 nm. The kinetic parameters *K*_m_ and *K*_cat_ were calculated by using the Hyper 32 program^3^, and the Lineweaver–Burk plot results were represented.

### Treatment of *G. biloba* Suspension Cells with SA and MeJA

Leaf-derived callis of *G. biloba* were obtained on MS solid medium supplied with 16 μM NAA, 4 μM 6-BA, 4 μM 2, 4-D, and 5 μM KT. The calli were transferred into liquid medium with the same hormones for cell suspension culture under a rotating speed of 100 rpm. The suspension cells were aliquoted and treated with 1 mM SA and 1 mM MeJA. After treatment, the cells were harvested at 2-, 4-, 8-, 12-, 24-, and 48-h after treatment, and freeze dried for further analyses.

### Ectopic Expression of *UGT716A1* in *A. thaliana*

The ORF of *UGT716A1* was ligated to the plant binary vector pCXSN (Chen et al., 2009), and introduced into *Agrobacterium* strain GV3101 for *A. thaliana* (Columbia-0) transformation using the floral dipping method (Clough and Bent, 1998). *A. thaliana* plants were grown at 22°C with 16 h/8 h light and dark cycles.

Total RNAs from the transgenic and wild type *A. thaliana* (Col-0) were extracted using Trizol-A^+^ reagent (Tiangen, China). cDNAs were synthesized by using reverse transcription with oligo primers (Promega, Germany) after DNase I treatment. Primes pairs UGT716A1XF/UGT716A1HR, and PP2A-F/PP2A-R were used in the RT-PCR. The PCR cycles for *UGT716A1* and *PP2A* were 35 and 33, respectively.

### Analyses of Total Flavonoids and Proanthocyanidins

Total flavonoids were extracted from leaves, stems, and roots of *G. biloba*, and 10-day-old *A. thaliana* seedlings (10 mg dry weight), with 500 μL 80% methanol. The flavonoid profiles were analyzed by HPLC with 50 μL extract using the same method as for enzymatic assay described above. For flavonoid quantification, each flavonoid compounds were relatively quantified based on a standard curve constructed with quercetin as standard. Proanthocyanidins were extracted from seeds of transgenic and wild type *A. thaliana* (20 mg) with 600 μL extraction buffer (70 % acetone with 0.5% acetic acid) three times. Total extractable PAs were quantified with the DMACA-based method and determined at wavelength at 640 nm, and non-extractable PAs were determined by butanol-HCl hydrolysis, and determined at wavelength of 550 nm as previously described (Pang et al., 2007).

### Accession Numbers

The GenBank accession numbers and plant species for different UGT protein sequences are: BvGT1, AAS94329 (*Beta vulgaris*); BvGT2, AAS94330 (*B. vulgaris*); Cs3GT, AAS00612 (*Citrus sinensis*); DicGT1, BAD52003 (*Dianthus caryophyllus*); DicGT3, BAD52005 (*D. caryophyllus*); FaGT6, ABB92748 (*Fragaria* × *ananassa*); GeIF7GT, BAC78438 (*Glycyrrhiza echinata*); GhA5GT, BAA36423 (*Glandularia* × *hybrida*); Mt7GT, AAW56091 (*Medicago truncatula*); MtUGT71G1, AAW56092 (*M. truncatula*); PfA5GT, BAA36421 (*Perilla frutescens var. crispa*); Ph3galT, AAD55985 (*Petunia* × *hybrida*); PhA5GT, BAA89009 (*P. hybrida*); RhGT4, BAE72453 (*Rosa hybrid*); Scb7GT, BAA83484 (*Scutellaria baicalensis*); SmGT, Q43641 (*Solanum melongena*); ThA5GT, BAC54093 (*Torenia hybrid*); UGT715A1, KX371618 (*G. biloba*); UGT716A1, KX371617 (*G. biloba*); UGT717A1, KX371619 (*G. biloba*); NtUGT71A11, BAB88934 (*Nicotiana tabacum*); NtUGT71A6, BAB60720 (*N. tabacum*); NtUGT71A7, BAB60721 (*N. tabacum*); Vv3GT, AAB81682 (*Vitis vinifera*); UGT721B1, KY274815 (*G. biloba*); UGT725A1, KY274816 (*G. biloba*); UGT726A1, KY274817 (*G. biloba*); UGT92K1, KY274818 (*G. biloba*); UGT725B1, KY274819 (*G. biloba*); UGT727A1, KY274820 (*G. biloba*); UGT73AS1, KY274821 (*G. biloba*).

## Author Contributions

XS and GS performed the experiments and analyzed the data. SD provided technical assistance to XS. XS drafted parts of the manuscript. RD interpreted data, revised the manuscript critically. YP conceived the project, supervised the experiments, and completed the writing.

## Conflict of Interest Statement

The authors declare that the research was conducted in the absence of any commercial or financial relationships that could be construed as a potential conflict of interest.

## Footnotes

1 http://www.cazy.org

2 http://medicinalplantgenomics.msu.edu/final_version_release_info.shtml

3 http://hyper32.software.informer.com/
