# Supplementary material for: Characterization of UGT716A1 as a Multi-substrate UDP:Flavonoid Glucosyltransferase Gene in Ginkgo biloba
Source: Front Plant Sci. 2017 Dec 7;8:2085. doi: 10.3389/fpls.2017.02085 (PMC5725826; doi:10.3389/fpls.2017.02085)
Supplement: Supplementary file 1 [file Image_1.PDF]

Supporting Information

Table S1. Main structural genes involved in the flavonoid biosynthetic pathway in the *G. biloba* leaf transcriptome

Abbreviations	Gene name	Number
C4H	cinnamate 4-hydroxylase	5
4CL	4-coumarate: CoA ligase	2
CHS	chalcone synthase	3
CHI	chalcone isomerase	3
F3H	flavanone 3-hydroxylase	13
FLS	flavonol synthase	11
UFGT	UDP:flavonoid glucosyltransferase	25

Table S2 List of 121 unigenes mentioned in this study

Unigene Number	Annotation
**comp635_c0_seq1_6**	ref|XP_002514595.1| UDP-glucosyltransferase, putative [*Ricinus communis*]
comp921_c0_seq1_8	ref|XP_002265438.2| PREDICTED: probable glycosyltransferase At5g03795-like [*Vitis vinifera*]
comp2583_c0_seq1_3	gb|AAD30619.1|AC007153_11 similar to indole-3-acetate beta-glucosyltransferase [*Arabidopsis thaliana*]
comp4509_c0_seq1_2	ref|XP_002510893.1| Xyloglucan galactosyltransferase KATAMARI1, putative [*Ricinus communis*]
**comp9346_c0_seq1_6**	ref|XP_003598165.1| Cytokinin-O-glucosyltransferase [*Medicago truncatula*]
comp11490_c0_seq1_2	ref|XP_002533436.1| glycosyltransferase, putative [*Ricinus communis*]
**comp11973_c0_seq1_6**	ref|XP_003632051.1| PREDICTED: UDP-glycosyltransferase 92A1-like [*Vitis vinifera*]
**comp14934_c0_seq1_3**	ref|XP_002518722.1| UDP-glucosyltransferase, putative [*Ricinus communis*]
**comp15453_c0_seq1_6**	ref|XP_002279299.1| PREDICTED: UDP-glycosyltransferase 83A1 [*Vitis vinifera*]
comp15578_c0_seq1_3	ref|XP_003602583.1| Secondary cell wall-related glycosyltransferase family [*Medicago truncatula*]
comp16803_c0_seq1_3	ref|YP_005679316.1| glycosyltransferase [*Clostridium botulinum H04402 065*]
comp17591_c0_seq1_38	dbj|BAA83484.1| UDP-glucose: flavonoid 7-O-glucosyltransferase [*Scutellaria baicalensis*]
comp18387_c0_seq1_34	ref|XP_003539326.1| PREDICTED: O-glucosyltransferase rumi-like [*Glycine max*]
comp18592_c0_seq1_22	ref|XP_003532702.1| PREDICTED: probable dolichyl pyrophosphate Glc1Man9GlcNAc2 alpha-1,3-glucosyltransferase-like [*Glycine max*]
comp18615_c0_seq1_13	ref|XP_003623063.1| Alpha-1,2-glucosyltransferase ALG10-A [*Medicago truncatula*]

Unigene Number	Annotation
**comp18915_c0_seq1_30**	ref|XP_002276808.1| PREDICTED: hydroquinone glucosyltransferase-like [*Vitis vinifera*]
comp18961_c0_seq1_11	ref|XP_002269459.2| PREDICTED: probable glycosyltransferase At5g03795-like [*Vitis vinifera*]
comp19071_c1_seq1_24	ref|XP_002965647.1| glycosyltransferase CAZy family 14 [*Selaginella moellendorffii*]
comp19569_c0_seq1_1	ref|XP_002324094.1| glycosyltransferase [*Populus trichocarpa*]
comp21946_c0_seq1_6	ref|XP_002960291.1| family 2 glycosyltransferase [*Selaginella moellendorffii*]
comp22797_c1_seq1_4	ref|XP_003633289.1| PREDICTED: xylosyltransferase 1-like [*Vitis vinifera*]
**comp23937_c0_seq1_4**	gb|AEQ33588.2| putative UDP-glucose:flavonoid glucosyltransferase [*Ginkgo biloba*]
comp23937_c1_seq1_2	;gb|AEQ33588.2| putative UDP-glucose:flavonoid glucosyltransferase [*Ginkgo biloba]*
**comp24628_c0_seq1_5**	ref|XP_002269003.2| PREDICTED: UDP-glycosyltransferase 74E2 [*Vitis vinifera*]
comp24649_c0_seq1_6	gb|EAX05587.1| UDP glucuronosyltransferase 2 family, polypeptide B11, isoform CRA_b [*Homo sapiens*]
comp24733_c0_seq1_7	ref|XP_002283615.1| PREDICTED: dolichyl-phosphate beta-glucosyltransferase [*Vitis vinifera*]
**comp24903_c0_seq1_38**	ref|XP_002276804.1| PREDICTED: UDP-glycosyltransferase 83A1 [*Vitis vinifera*]
**comp25088_c0_seq1_13**	ref|XP_002519853.1| UDP-glucosyltransferase, putative [*Ricinus communis*]
comp25164_c0_seq1_12	ref|XP_002273962.2| PREDICTED: galacturonosyltransferase 8-like [*Vitis vinifera*]
comp25279_c0_seq1_33	ref|XP_002978152.1| glycosyltransferase CAZy family GT34-like protein [*Selaginella moellendorffii*]
comp25461_c0_seq1_16	ref|XP_002280181.1| PREDICTED: probable dolichyl pyrophosphate Man9GlcNAc2 alpha-1,3-glucosyltransferase [*Vitis vinifera*]

Unigene Number	Annotation
comp25817_c0_seq1_38	ref|XP_002276440.2| PREDICTED: probable glycosyltransferase At5g11130-like [*Vitis vinifera*]
comp26363_c0_seq1_14	ref|XP_002268876.2| PREDICTED: probable glycosyltransferase At5g03795-like [*Vitis vinifera*]
comp26363_c1_seq1_6	ref|XP_002268876.2| PREDICTED: probable glycosyltransferase At5g03795-like [*Vitis vinifera*]
comp26409_c0_seq1_2	ref|NP_001059081.1| Os07g0188700 [*Oryza sativa Japonica Group*] dbj|BAC83913.1| putative pectin-glucuronyltransferase
comp26409_c1_seq1_4	ref|XP_002264111.2| PREDICTED: probable glycosyltransferase At3g07620-like [*Vitis vinifera*]
comp26774_c0_seq1_15	gb|AEQ33588.2| putative UDP-glucose:flavonoid glucosyltransferase [*Ginkgo biloba*]
comp28332_c0_seq1_10	ref|YP_401098.1| glycosyl transferase [*Synechococcus elongatus PCC 7942*]
comp28636_c0_seq1_21	ref|XP_003552954.1| PREDICTED: O-glucosyltransferase rumi-like [*Glycine max*]
comp28932_c0_seq1_6	ref|XP_002963550.1| family 2 glycosyltransferase [*Selaginella moellendorffii*]
comp28932_c2_seq1_13	ref|XP_002983952.1| family 2 glycosyltransferase [*Selaginella moellendorffii*]
comp29392_c1_seq1_3	gb|AEQ33588.2| putative UDP-glucose:flavonoid glucosyltransferase [*Ginkgo biloba*]
comp29345_c0_seq1_20	ref|XP_002989750.1| glycosyltransferase belonging to CAZy family GT61 [*Selaginella moellendorffii*]
comp29345_c1_seq1_8	ref|XP_002989750.1| glycosyltransferase belonging to CAZy family GT61 [*Selaginella moellendorffii*]
comp29361_c0_seq1_4	ref|XP_003521167.1| PREDICTED: uncharacterized glycosyltransferase AGO61-like [*Glycine max*]
comp29361_c1_seq1_8	gb|ACG27644.1| glycosyltransferase [*Zea mays*]
comp29392_c0_seq1_5	gb|AEQ33588.2| putative UDP-glucose:flavonoid glucosyltransferase [*Ginkgo biloba*]

Unigene Number	Annotation
**comp29490_c3_seq1_5**	gb|AAD30619.1|AC007153_11 similar to indole-3-acetate beta-glucosyltransferase [*Arabidopsis thaliana*]
**comp29604_c0_seq1_3**	dbj|BAK55737.1| UDP-glucose glucosyltransferase [*Gardenia jasminoides*]
comp29771_c1_seq1_4	ref|XP_001778678.1| cellulose synthase-like D7, glycosyltransferase family 2
comp29877_c0_seq1_19	ref|XP_002281658.1| PREDICTED: probable galacturonosyltransferase-like 3 [*Vitis vinifera*]
comp30089_c0_seq1_56	gb|ABS70459.1| alpha-1,2-fucosyltransferase [*Populus tremula x Populus alba*]
comp30482_c0_seq1_18	ref|XP_003516821.1| PREDICTED: dolichyl-diphosphooligosaccharide--protein glycosyltransferase subunit STT3-like [*Glycine max*]
comp30482_c1_seq1_15	ref|XP_003516821.1| PREDICTED: dolichyl-diphosphooligosaccharide--protein glycosyltransferase subunit STT3-like [*Glycine max*]
comp31083_c0_seq1_80	ref|XP_002268972.1| PREDICTED: UDP-glucose:glycoprotein glucosyltransferase-like [*Vitis vinifera*]
comp31253_c0_seq1_10	AltName: Full=Sucrose-UDP glucosyltransferase 2 emb|CAA04512.1| second sucrose synthase [*Pisum sativum*]
comp31400_c1_seq1_26	gb|AEQ33588.2| putative UDP-glucose:flavonoid glucosyltransferase [*Ginkgo biloba*]
comp31489_c0_seq1_7	ref|XP_003552555.1| PREDICTED: probable galacturonosyltransferase 13-like [*Glycine max*]
comp31489_c2_seq1_8	ref|XP_002324094.1| glycosyltransferase [*Populus trichocarpa*]
comp31489_c4_seq1_4	ref|XP_002983680.1| galacturonosyltransferase GAUT12/13/14/15-like protein [*Selaginella moellendorffii*]
**comp31613_c1_seq1_45**	ref|NP_568137.1| UDP-glycosyltransferase family protein [*Arabidopsis thaliana*]
comp31894_c0_seq1_7	gb|AED99886.1| glycosyltransferase [*Panax notoginseng*]
comp32700_c0_seq1_12	gb|AEQ33588.2| putative UDP-glucose:flavonoid glucosyltransferase [*Ginkgo biloba*]

Unigene Number	Annotation
comp32745_c0_seq1_5	ref|XP_002965647.1| glycosyltransferase CAZy family 14 [*Selaginella moellendorffii*]
comp32804_c0_seq1_12	ref|XP_003531191.1| PREDICTED: probable glycosyltransferase At3g07620-like isoform 1 [*Glycine max*]
comp33780_c0_seq1_40	ref|XP_002268382.1| PREDICTED: xylogalacturonan beta-1,3-xylosyltransferase [*Vitis vinifera*]
**comp34006_c0_seq1_11**	ref|XP_002268242.2| PREDICTED: UDP-glycosyltransferase 85A3 [*Vitis vinifera*]
comp34188_c0_seq1_44	gb|EFJ38389.1| glycosyltransferase CAZy family GT14 [*Selaginella moellendorffii*]
comp34713_c0_seq1_14	gb|AEQ33588.2| putative UDP-glucose:flavonoid glucosyltransferase [*Ginkgo biloba*]
comp34749_c2_seq1_13	ref|XP_002960850.1| glycosyltransferase CAZy family GT14 [*Selaginella moellendorffii*]
comp35143_c0_seq1_8	ref|XP_003537048.1| PREDICTED: dolichyl-diphosphooligosaccharide--protein glycosyltransferase subunit 2-like [*Glycine max*]
comp35414_c0_seq1_12	ref|XP_002514720.1| Xyloglucan 6-xylosyltransferase, putative [*Ricinus communis*]
comp35899_c0_seq1_2	ref|XP_002320745.1| glycosyltransferase [*Populus trichocarpa*]
comp35899_c2_seq1_10	ref|XP_003535002.1| PREDICTED: probable galacturonosyltransferase 4-like isoform 1 [*Glycine max*]
comp36003_c0_seq1_18	ref|XP_003527283.1| PREDICTED: dolichyl-diphosphooligosaccharide--protein glycosyltransferase subunit 1-like [*Glycine max*]
comp36564_c0_seq1_6	ref|XP_002265312.1| PREDICTED: UDP-sugar-dependent glycosyltransferase 52-like [*Vitis vinifera*]
comp36564_c1_seq1_22	ref|XP_002265312.1| PREDICTED: UDP-sugar-dependent glycosyltransferase 52-like [*Vitis vinifera*]
comp36564_c2_seq1_5	ref|XP_003535566.1| PREDICTED: UDP-sugar-dependent glycosyltransferase 52-like [*Glycine max*]
comp37029_c1_seq1_35	ref|XP_002283249.1| PREDICTED: probable beta-1,4-xylosyltransferase IRX14H-like [*Vitis vinifera*]

Unigene Number	Annotation
comp37053_c0_seq1_15	ref|XP_003635418.1| PREDICTED: probable xyloglucan glycosyltransferase 6-like [*Vitis vinifera*]
comp37053_c1_seq1_4	ref|XP_003558757.1| PREDICTED: probable xyloglucan glycosyltransferase 9-like [*Brachypodium distachyon*]
comp37053_c2_seq1_28	ref|XP_002988944.1| cellulose synthase-like C1-2, glycosyltransferase family 2 protein
comp37071_c0_seq1_21	ref|XP_002280923.1| PREDICTED: hydroquinone glucosyltransferase [*Vitis vinifera*]
comp37283_c2_seq1_20	gb|AAX33322.1| secondary cell wall-related glycosyltransferase family 47
comp37437_c0_seq1_17	ref|XP_002969509.1| Glycosyltransferase, CAZy family GT8 [*Selaginella moellendorffii*]
comp37465_c0_seq1_40	ref|XP_002970865.1| Glycosyltransferase, CAZy family GT8 [*Selaginella moellendorffii*]
comp37631_c1_seq1_30	dbj|BAM29304.1| acyl-glucose-dependent anthocyanin 7-O-glucosyltransferase [*Agapanthus africanus*]
comp37655_c3_seq1_5	ref|XP_002983952.1| family 2 glycosyltransferase [*Selaginella moellendorffii*]
comp37887_c0_seq1_14	gb|EEF37195.1| Glycosyltransferase QUASIMODO1, putative [*Ricinus communis*]
comp37887_c1_seq1_19	ref|XP_002525229.1| Glycosyltransferase QUASIMODO1, putative
**comp37969_c1_seq1_10**	ref|XP_003632051.1| PREDICTED: UDP-glycosyltransferase 92A1-like [*Vitis vinifera*]
**comp38122_c0_seq1_15**	ref|XP_002276804.1| PREDICTED: UDP-glycosyltransferase 83A1 [*Vitis vinifera*]
comp38192_c0_seq1_36	gb|AEQ33588.2| putative UDP-glucose:flavonoid glucosyltransferase [*Ginkgo biloba*]
comp38192_c0_seq1_36	gb|AEQ33588.2| putative UDP-glucose:flavonoid glucosyltransferase [*Ginkgo biloba*]
comp38257_c0_seq1_10	gb|AEQ33588.2| putative UDP-glucose:flavonoid glucosyltransferase [*Ginkgo biloba*]

Unigene Number	Annotation
comp38289_c0_seq1_16	ref|XP_002271933.1| PREDICTED: probable xyloglucan glycosyltransferase 5 [*Vitis vinifera*]
comp38289_c1_seq1_8	;ref|XP_002271933.1| PREDICTED: probable xyloglucan glycosyltransferase 5 [*Vitis vinifera*]
comp38289_c3_seq1_42	ref|XP_002271933.1| PREDICTED: probable xyloglucan glycosyltransferase 5 [*Vitis vinifera*]
comp38405_c0_seq1_8	ref|XP_002284381.1| PREDICTED: zeatin O-glucosyltransferase-like [*Vitis vinifera*]
**comp38556_c0_seq1_15**	ref|XP_003519477.1| PREDICTED: UDP-glycosyltransferase 92A1-like [*Glycine max*]
**comp38591_c3_seq1_13**	ref|XP_003632262.1| PREDICTED: UDP-glycosyltransferase 86A1-like [*Vitis vinifera*]
comp38852_c0_seq1_13	ref|XP_002263821.2| PREDICTED: putative glycosyltransferase 2 [*Vitis vinifera*]
comp39628_c0_seq1_9	ref|XP_002284627.1| PREDICTED: dolichyl-diphosphooligosaccharide--protein glycosyltransferase 48 kDa subunit [*Vitis vinifera*]
comp39764_c0_seq1_27	ref|XP_002269119.2| PREDICTED: dolichyl-diphosphooligosaccharide--protein glycosyltransferase subunit STT3-like [*Vitis vinifera*]
comp51907_c0_seq1_10	gb|AAK73021.1|AF367245_1 ceramide glucosyltransferase [*Gossypium arboreum*]
comp64886_c0_seq1_11	ref|XP_003517290.1| PREDICTED: probable glycosyltransferase At5g03795-like [*Glycine max*]
comp64980_c0_seq1_9	gb|AAB36653.1| immediate-early salicylate-induced glucosyltransferase [*Nicotiana tabacum*]
**comp75899_c0_seq1_29**	ref|XP_002268162.1| PREDICTED: UDP-glycosyltransferase 85A2 [*Vitis vinifera*]
**comp103445_c0_seq1_19**	gb|ABL85472.1| glycosyltransferase UGT72B9 [*Maclura pomifera*]
comp116323_c0_seq1_2	ref|YP_005311452.1| glycosyltransferase [*Paenibacillus mucilaginosus 3016*]
comp142051_c0_seq1_5	ref|ZP_10971454.1| glycosyl transferase family protein [*Sporolactobacillus vineae DSM 21990 = SL153*]

Unigene Number	Annotation
**comp143607_c0_seq1_2**	ref|NP_181218.1| cytokinin-O-glucosyltransferase 3 [*Arabidopsis thaliana*]
comp144529_c0_seq1_11	gb|AEQ33588.2| putative UDP-glucose:flavonoid glucosyltransferase [*Ginkgo biloba*]
comp194550_c0_seq1_1	gb|AAM15782.1|AC104428_3 Putative indole-3-acetate beta-glucosyltransferase [*Oryza sativa Japonica Group*]
comp198906_c0_seq1_5	ref|XP_003560175.1| PREDICTED: anthocyanidin 5,3-O-glucosyltransferase-like [*Brachypodium distachyon*]
comp206883_c0_seq1_4	ref|YP_003778631.1| glycosyltransferase [*Clostridium ljungdahlii DSM 13528*]
**comp215683_c0_seq1_16**	ref|NP_001190649.1| hydroquinone glucosyltransferase [*Arabidopsis thaliana*]
**comp263434_c0_seq1_1**	ref|XP_002276825.1| PREDICTED: UDP-glycosyltransferase 83A1 [*Vitis vinifera*]
comp277444_c0_seq1_10	ref|XP_003554026.1| PREDICTED: probable glycosyltransferase At5g03795-like [*Glycine max*]
**comp283902_c0_seq1_2**	ref|NP_001148083.1| UDP-glycosyltransferase/ transferase, transferring glycosyl groups
**comp310134_c0_seq1_2**	ref|XP_002513452.1| UDP-glucosyltransferase, putative [*Ricinus communis*]

Note: Unigenes mentioned in this study were in bold.

Table S3. Twenty-five *UFGT* unigenes identified in the *G. biloba* leaf transcriptome

Unigene Number	Length (nt)	Nomenclature	ORF length （bp）	Protein length
comp635_c0_seq1_6	222			
comp9346_c0_seq1_6	312			
comp11973_c0_seq1_6	231			
**comp14934_c0_seq1_3**	**332**	**UGT715A1**	**1281**	**426**
comp15453_c0_seq1_6	345			
comp18915_c0_seq1_30	1512			
**comp23937_c0_seq1_4**	**465**	**UGT721B1**	**1500**	**499**
comp24628_c0_seq1_5	264			
**comp24903_c0_seq1_38**	**1455**	**UGT725A1**	**1446**	**481**
**comp25088_c0_seq1_13**	**1593**	**UGT716A1**	**1539**	**512**
comp29490_c3_seq1_5	312			
comp29604_c2_seq1_2	219			
**comp310134_c0_seq1_2**	**204**	**UGT717A1**	**1377**	**458**
comp31613_c1_seq1_45	2091			
**comp34006_c0_seq1_11**	**1500**	**UGT726A1**	**1422**	**473**
**comp37969_c1_seq1_10**	**1101**	**UGT92K1**	**1491**	**496**
**comp38122_c0_seq1_15**	**927**	**UGT725B1**	**1440**	**479**
comp38556_c0_seq1_15	1512			
comp38591_c3_seq1_13	678			
comp75899_c0_seq1_29	1449			
**comp103445_c0_seq1_19**	**774**	**UGT727A1**	**1521**	**506**
**comp143607_c0_seq1_2**	**489**	**UGT727A1**	**1521**	**506**
**comp215683_c0_seq1_16**	**726**	**UGT73AS1**	**1455**	**484**
**comp263434_c0_seq1_1**	**219**	**UGT725A1**	**1446**	**481**
comp283902_c0_seq1_2	225			

Table S4. Sequence identity between the deduced GbUGT proteins at the amino acid level

	UGT715A1	UGT716A1	UGT717A1	UGT721B1	UGT725A1	UGT725B1	UGT726A1	UGT727A1	UGT73AS1	UGT92K1
**UGT715A1**	-	35%	37%	31%	30%	30%	32%	36%	34%	43%
**UGT716A1**	-	-	44%	30%	38%	26%	37%	33%	33%	34%
**UGT717A1**	-	-	-	31%	28%	29%	29%	34%	33%	39%
**UGT721B1**	-	-	-	-	28%	29%	28%	31%	29%	36%
**UGT725A1**	-	-	-	-	-	61%	36%	33%	30%	32%
**UGT725B1**	-	-	-	-	-	-	36%	37%	32%	34%
**UGT726A1**	-	-	-	-	-	-	-	31%	31%	32%
**UGT727A1**	-	-	-	-	-	-	-	-	34%	40%
**UGT73AS1**	-	-	-	-	-	-	-	-	-	33%
**UGT92K1**	-	-	-	-	-	-	-	-	-	-

Table S5. Primer sequences used in the present study

Primers	Sequence (5'-3', the corresponding restriction sites were underlined)	Note	Restriction sites
UGT715A1BF	CGGGATCCATGGGGAAGATAAAGAGCCCTC	For gene cloning	*Bam* H Ⅰ
UGT715A1SR	GCGTCGACTCATGTCTGTTTCTTTGGGTATG	For gene cloning	*Sal* Ⅰ
UGT716A1XF	GCTCTAGAATGGAAATGGCTGGAGGTTAC	For gene cloning	*Xba* Ⅰ
UGT716A1HR	CCCAAGCTTCTAGAGTTTCTTTTGCAAGC	For gene cloning	*Hind* Ⅲ
UGT717A1BF	CGGGATCCATGGAGAAGAGGAGGGGGCATG	For gene cloning	*Bam* H Ⅰ
UGT717A1SR	GCGTCGACTTAAAGACTACAATCTGCAGATG	For gene cloning	*Sal* Ⅰ
UGT721B1BF	CGGGATCCATGTCTATCATGGAGTTGTCTT	For gene cloning	*Bam* H Ⅰ
UGT721B1SR	ACGCGTCGACAATTCTAAGGCTCTAAACTATAT	For gene cloning	*Sal* Ⅰ
UGT725A1BF	CGGGATCCGTGAGCATTAAAATGGGTTTCA	For gene cloning	*Bam* H Ⅰ
UGT725A1SR	ACGCGTCGACTCAACTGTTCTTCGTGGCATT	For gene cloning	*Sal* Ⅰ
UGT726A1BF	CGGGATCCATGGCCAAGCAACCCCATGC	For gene cloning	*Bam* H Ⅰ
UGT726A1SR	ACGCGTCGACTTCCGATTGTCTCAATTCTTCATA	For gene cloning	*Sal* Ⅰ
UGT727A1BF	CGGGATCCATGGCGCCTGGCATAGAAC	For gene cloning	*Bam* H Ⅰ
UGT727A1SR	ACGCGTCGACTCATTGCTCTTCAAGAGCAAG	For gene cloning	*Sal* Ⅰ
UGT92K1BF	CGGGATCCATGGCTGCAGGCTCTCAGG	For gene cloning	*Bam* H Ⅰ
UGT92K1SR	ACGCGTCGACCTAGCCCTGGTTCTCCATGC	For gene cloning	*Sal* Ⅰ
PpUGT1XF	GCTCTAGAATGACCGTTGCCAGGCGAGA	For gene cloning	*Xba* Ⅰ
PpUGT1PR	AACTGCAGCTACAATCCGTTGGGTAGTTGT	For gene cloning	*Pst* Ⅰ
PpUGT2EF	CGGAATTCATGGGCTCCGAGGATCGTGT	For gene cloning	*EcoR* Ⅰ
PpUGT2XR	GCTCTAGATCAAGGGAGAGTTTTGAGGTG	For gene cloning	*Xba* Ⅰ
PpUGT3XF	GCTCTAGAATGGGTTCGATCGCGATGGAG	For gene cloning	*Xba* Ⅰ
PpUGT3HR	CCCAAGCTTCTAGAAGCTCGGCATTGGATTG	For gene cloning	*Hind*Ⅲ
VvUGT1XF	GCTCTAGAATGGAACACCCAACACCTC	For gene cloning	*Xba* Ⅰ
VvUGT1HR	CCCAAGCTTTCAAACCTTGATATTCTTCCA	For gene cloning	*Hind* Ⅲ

VvUGT3XF	GCTCTAGAATGTCTGTCCATATCATAGTG	For gene cloning	*Xba* Ⅰ
VvUGT3HR	CCCAAGCTTTTATGGAGCCCTTTGGTTGA	For gene cloning	*Hind* Ⅲ
MtUGT1BF	CGGGATCCATGTCTCAAGAAATTTGCATAC	For gene cloning	*Bam* H Ⅰ
MtUGT1SR	ACGCGTCGACCTAAACAAATCTCTGTTTGATAA	For gene cloning	*Sal* Ⅰ
MtUGT2BF	CGGGATCCATGACTAACGAAAATCAAGAACT	For gene cloning	*Bam* H Ⅰ
MtUGT2SR	ACGCGTCGACTTAGTATGCACGTGACTTCAAA	For gene cloning	*Sal* Ⅰ
UGT716A1RTF	AGAGCTCGCATATGGATTGG	For RT-PCR	-
UGT716A1RTR	CGGTTGATGGGTGAGAGAGT	For RT-PCR	-
PP2A-F	TATCGGATGACGATTCTTCGTGCAG	For RT-PCR	-
PP2A-R	GCTTGGTCGACTATCGGAATGAGAG	For RT-PCR	-

Table S6. Summary of the substrates tested in the present study

Names	Substrates	UGT715A1	UGT716A1	UGT717A1	UGT725A1	UGT725B1	UGT726A1	UGT727A1	UGT92K1
Anthocyanidins	pelargonidin	–	–	–	–	–	–	–	–
	cyanidin	–	–	–	–	–	–	–	–
	delphinidin	–	–	–	–	–	–	–	–
Flavonols	kaempferol	–	+	–	–	–	–	–	–
	quercetin	–	+	–	–	–	–	–	–
	myricetin	–	+	–	–	–	–	–	–
Flavones	apigenin	–	+	–	–	–	–	–	–
	luteolin	–	+	–	–	–	–	–	–
	tricetin	–	+	–	–	–	–	–	–
Isoflavones	daidzein	–	–	–	–	–	–	–	–
	genistein	–	+	–	–	–	–	–	+
(epi)-catechins	catechin	–	–	–	–	–	–	–	–
	epicatechin	–	–	–	–	–	–	–	–
	gallocatechin	–	–	–	–	–	–	–	–
	epigallocatechin	–	–	–	–	–	–	–	–
(epi)-(gallo)-catechin gallates	catechin gallate	–	+	–	–	–	–	–	–
	gallocatechin gallate	–	+	–	–	–	–	–	–
	epicatechin gallate	–	+	–	–	–	–	–	–
	epigallocatechin gallate	–	+	–	–	–	–	–	–
Gallic acids	gallic acid	–	+	–	–	–	–	–	–
	methyl gallic acid	–	+	–	–	–	–	–	–

Note: +, product was detected with UDP-glucose as sugar donor; –, no product was detected with UDP-glucose as sugar donor.

Table S7. Enzymatic activity of recombinant UGT716A1 protein toward various substrates with UDP-galactose as sugar donor.

**Substrates**	**Convrsion rate (%）**	**Substrates**	**Conversion rate（%）**
Kaempferol	3.0 ±0.3	Catechin gallate	0.3 ±0.0
Quercetin	20.7 ±1.2	Epicatechin gallate	25.1 ±1.5
Myricetin	18.4 ±1.4	Gallocatechin gallate	3.3 ±0.8
Apigenin	2.1 ±0.4	Epigallocatechin gallate	19.0 ±1.2
Lutiolin	33.8 ±1.7	Methyl gallic acid	0.9 ±0.0
Tricetin	39.4 ±1.8		

Values indicate the means from triplicate assays.

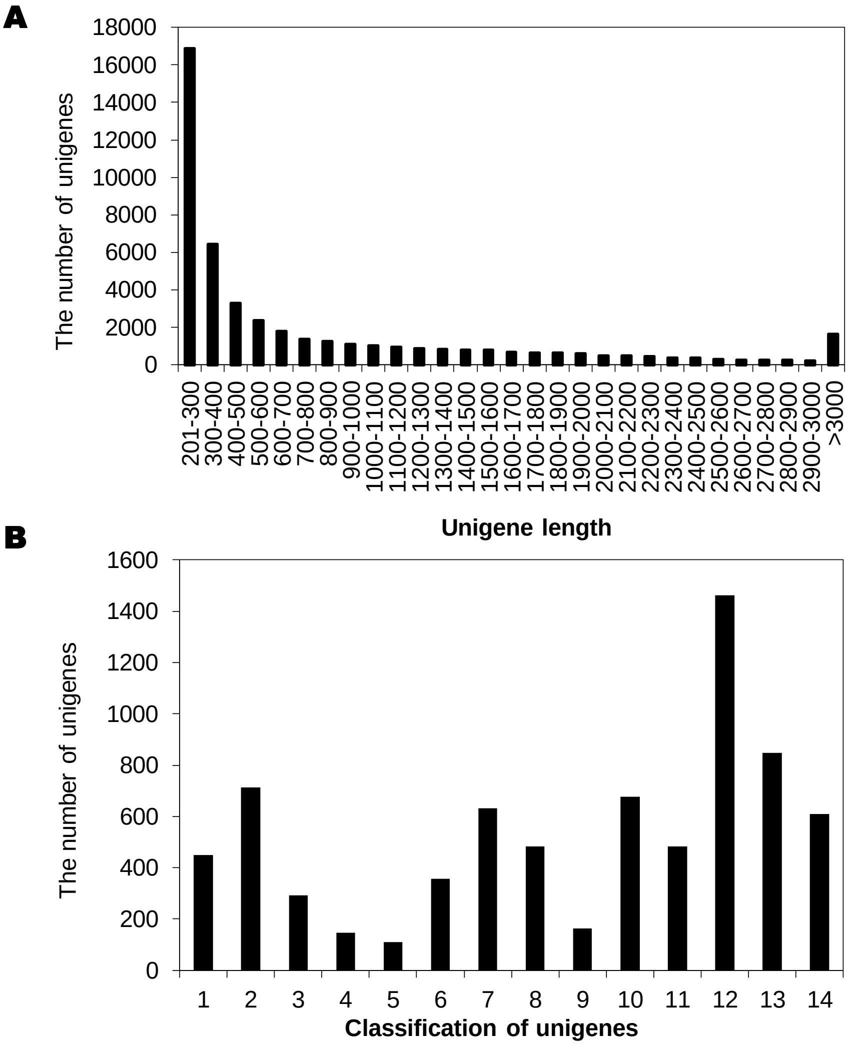

1 Amino acid transport and metabolism
2 Carbohydrate transport and metabolism
3 Cell cycle control, cell division, chromosome partitioning
4 Coenzyme transport and metabolism
5 Defense mechanisms
6 Inorganic ion transport and metabolism
7 Intracellular trafficking, secretion, and vesicular transport
8 Lipid transport and metabolism
9 Nucleotide transport and metabolism
10 RNA processing and modification
11 Secondary metabolites biosynthesis, transport and catabolism
12 Signal transduction mechanisms
13 Transcription
14 Translation, ribosomal structure and biogenesis

Figure S1. Analyses of the *G. biloba* leaf transcriptome.

(A) Length distribution of unigenes.

(B) Function classification of unigenes by KOG (euKaryotic Ortholog Groups).

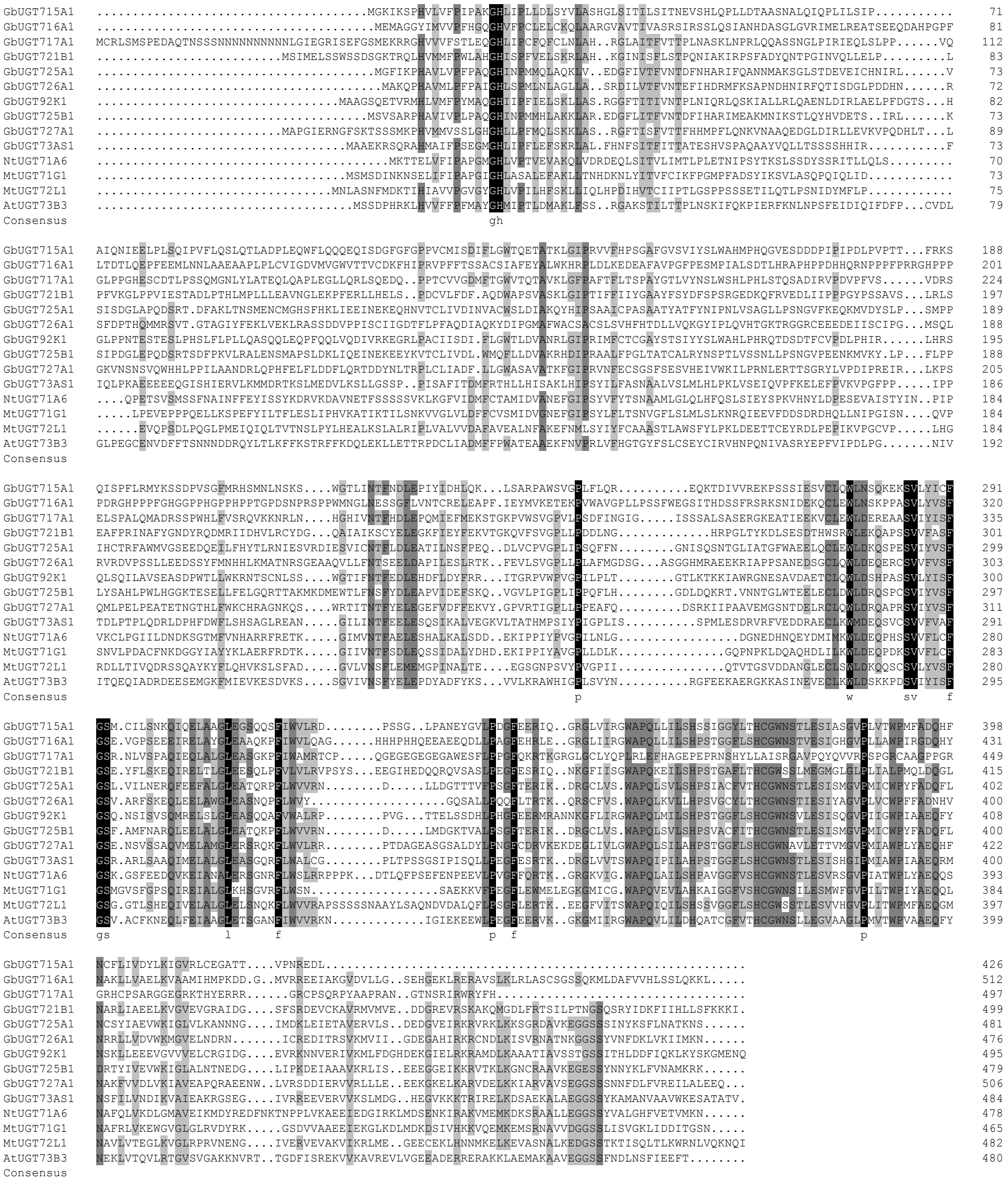

Figure S2. Multiple sequence alignment of GbUGTs with other functionally characterized UGTs.

Multiple sequence alignment of the ten UGTs in *G. biloba* with other functionally characterized UGTs. NtUGT71A6, BAB60720 (*Nicotiana tabacum*); MtUGT71G1, AAW56092 (*M. truncatula*); MtUGT72L1, ACC38470 (*M. truncatula*); AtUGT73B3, Q8W491.1 (*A. thaliana*).

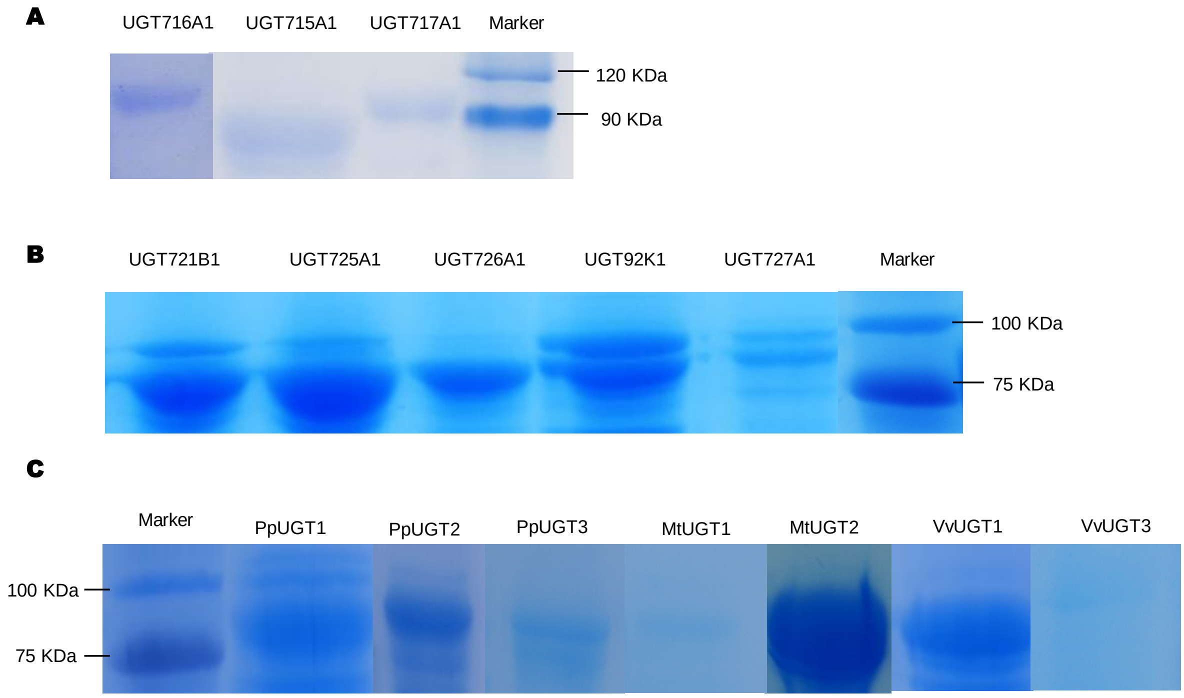

Figure S3. Purified recombinant UGT proteins detected on 12% SDS-PAGE.

(A) The recombinant UGT715A1, UGT716A1 and UGT717A1 proteins;

(B) The recombinant UGT721B1, UGT725A, UGT726A1, UGT92K1, UGT727A1 proteins.

(C) The recombinant PpUGT1, PpUGT2, PpUGT3, MtUGT1, MtUGT2, VvUGT1 and VvUGT3 proteins.

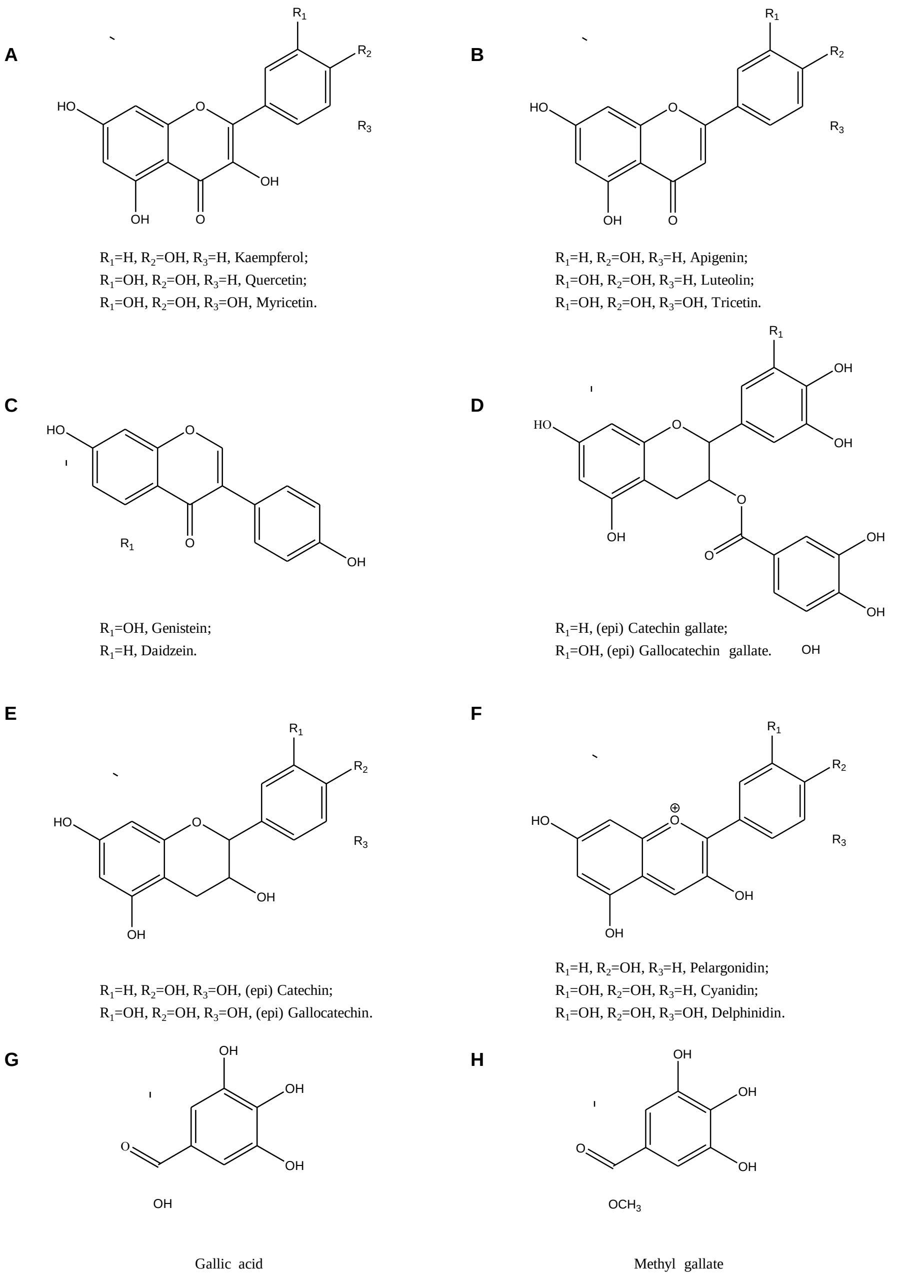

Figure S4 Representative chemical structures of flavonoid substrates tested in the present study.

(A) Flavonols; (B) Flavones; (C) Isoflavonoids; (D) (epi)-(gallo)-catechin gallates; (E) (epi)-(gallo)-catechin; (F) Anthocyanins; (G) Gallic acid; (H) Methyl gallate.

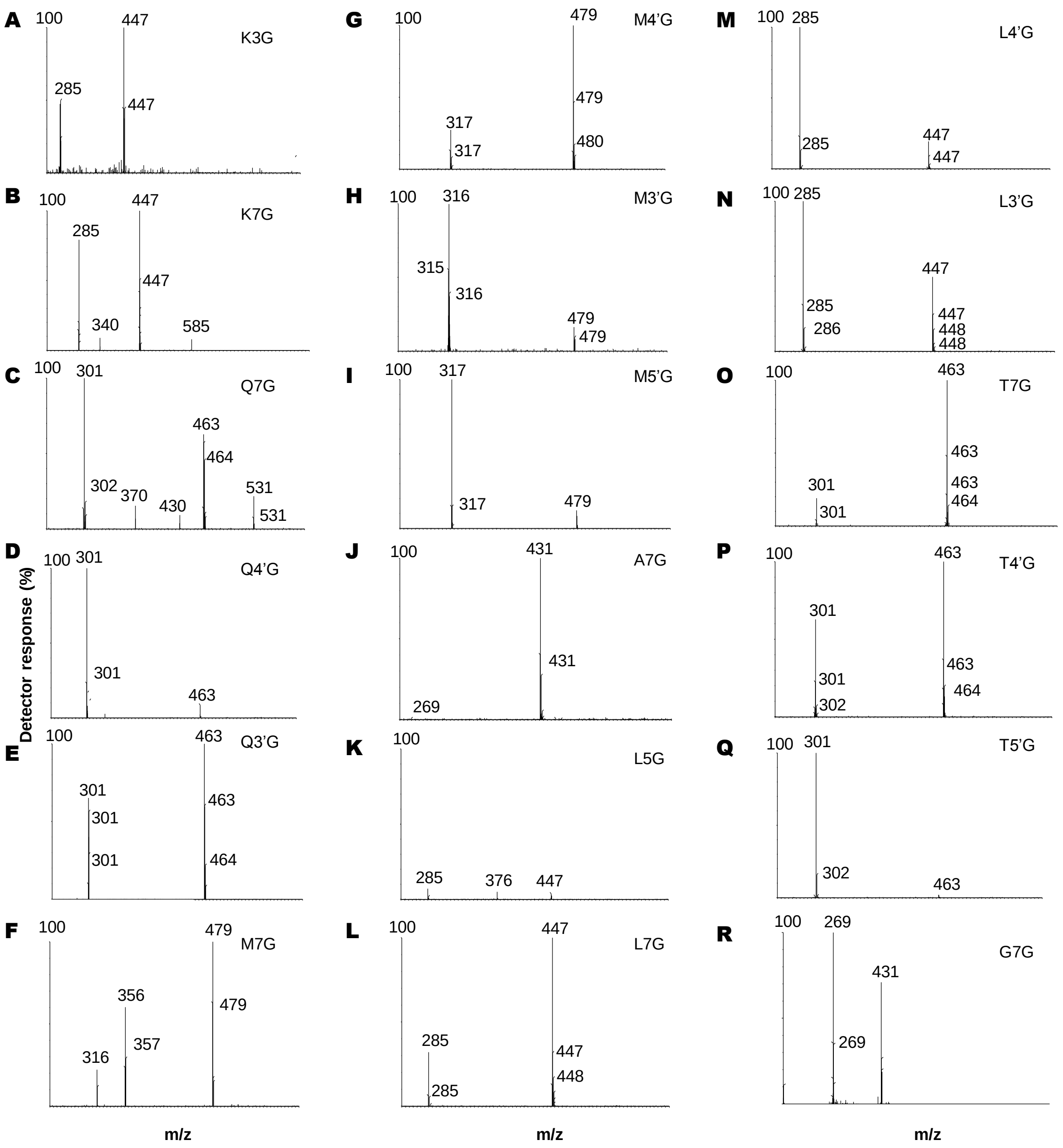

Figure S5. Mass spectra of the enzymatic products of recombinant UGT716A1 protein with flavonols, flavones and isoflavones as substrates.

(A-M) Mass spectrum of enzymatic products of the recombinant UGT716A1 protein with substrates of kaempferol (A-B), quercetin (C-E), myricetin (F-I), apigenin (J), luteolin (K-N), tricetin (O-Q) and genistein (R).

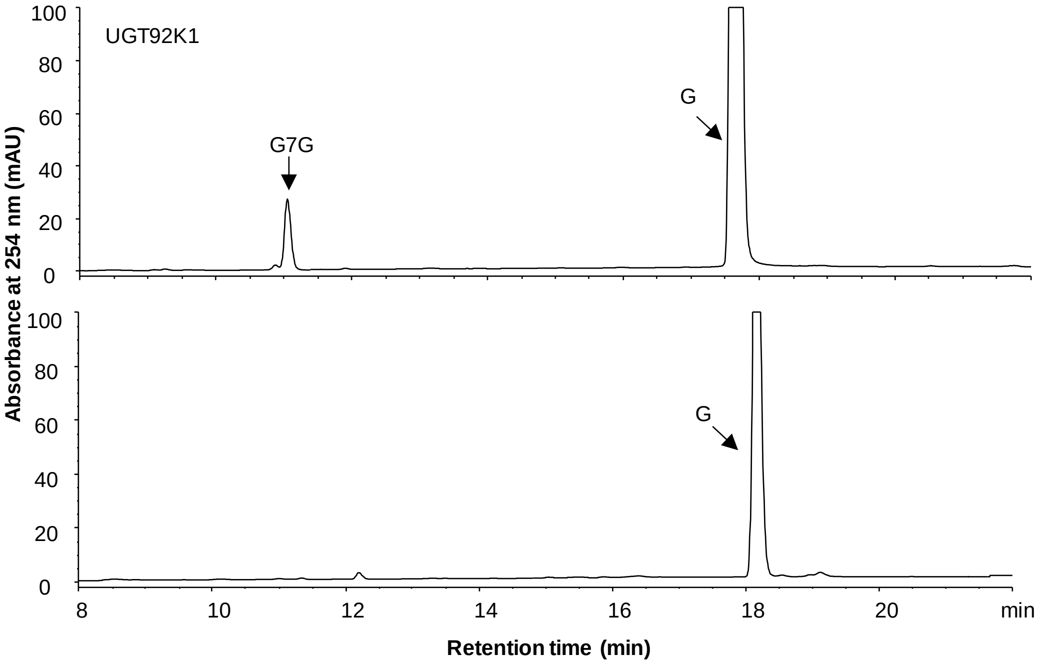

Figure S6. Analyses of the enzymatic product of recombinant UGT92K1 protein with genistein as substrate.

HPLC chromatograms of enzymatic products from enzymatic assays of the recombinant UGT92K1 protein (upper panels) and control (lower panels) with genistein substrate.

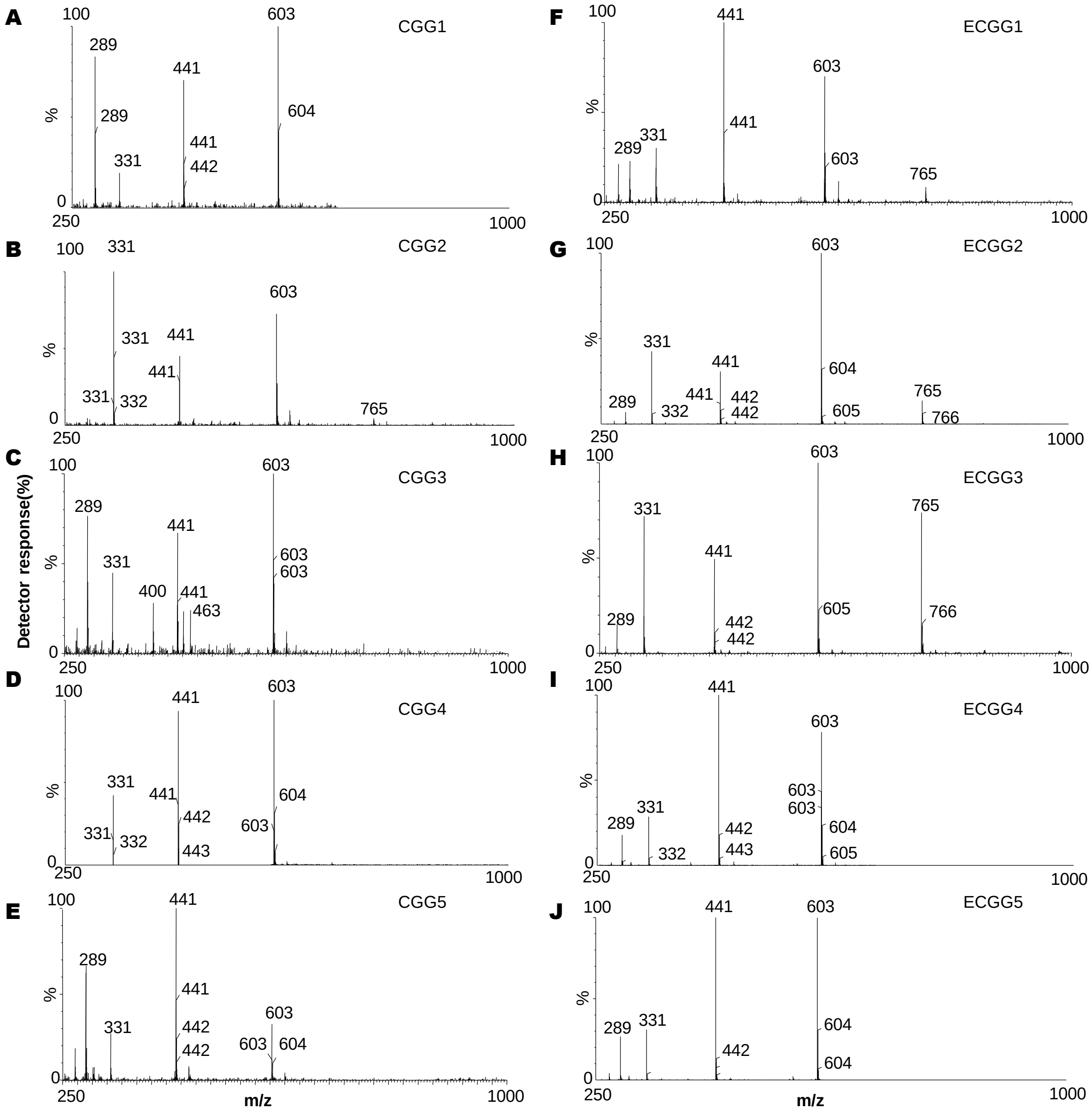

Figure S7. Mass spectra of the enzymatic products of recombinant UGT716A1 with (epi)-catechin gallates as substrates.

(A-J) Mass spectrum of enzymatic products of the recombinant UGT716A1 protein with substrates of catechin gallate (A-E) and epicatechin gallate (F-J).

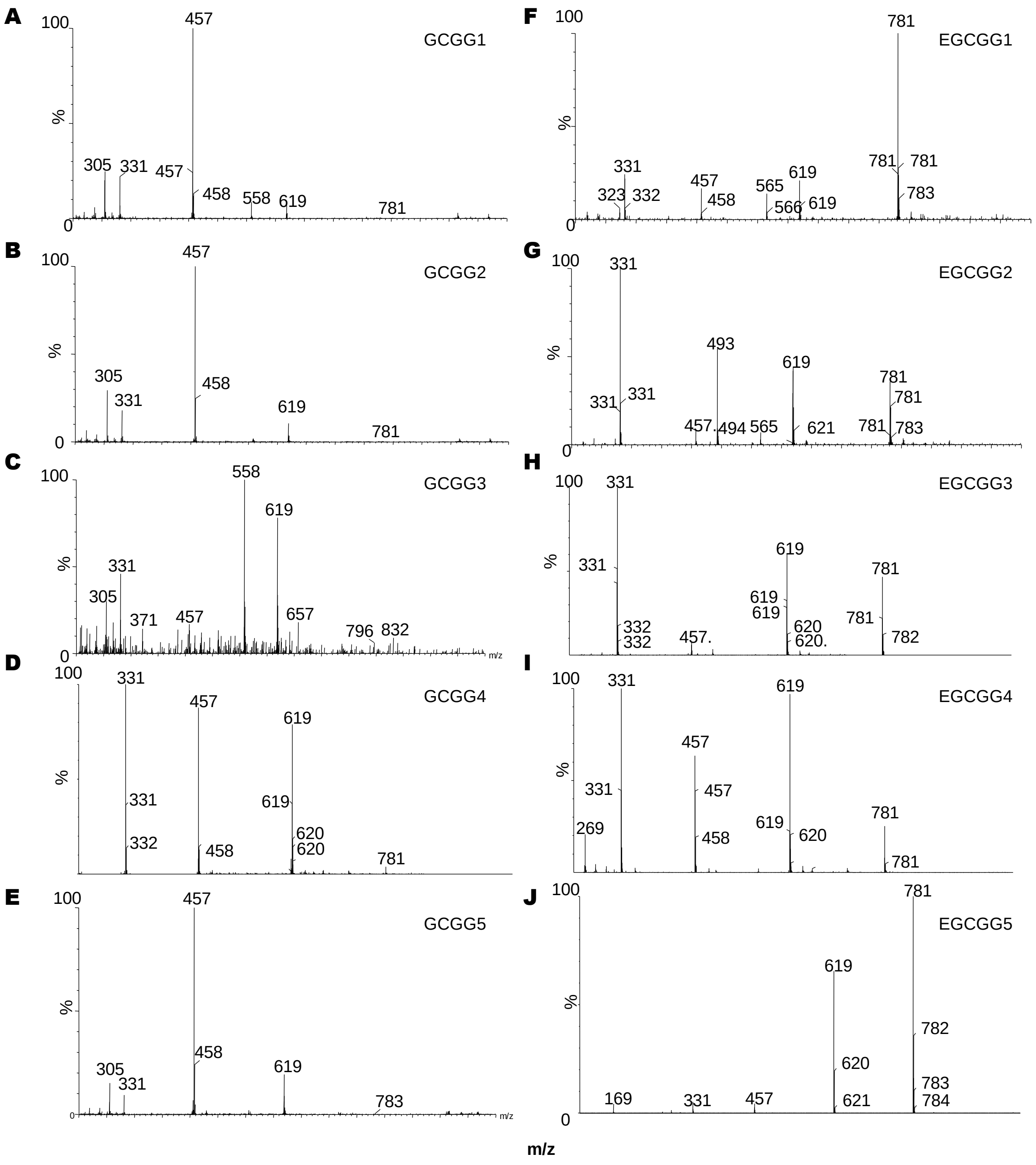

Figure S8. Mass spectra of the enzymatic products of recombinant UGT716A1 with (epi)-(gallo)-catechin gallates as substrates.

(A-J) Mass spectrum of enzymatic products of the recombinant UGT716A1 protein with substrates gallocatechin gallate (A-E) and epigallocatechin gallate (F-J).

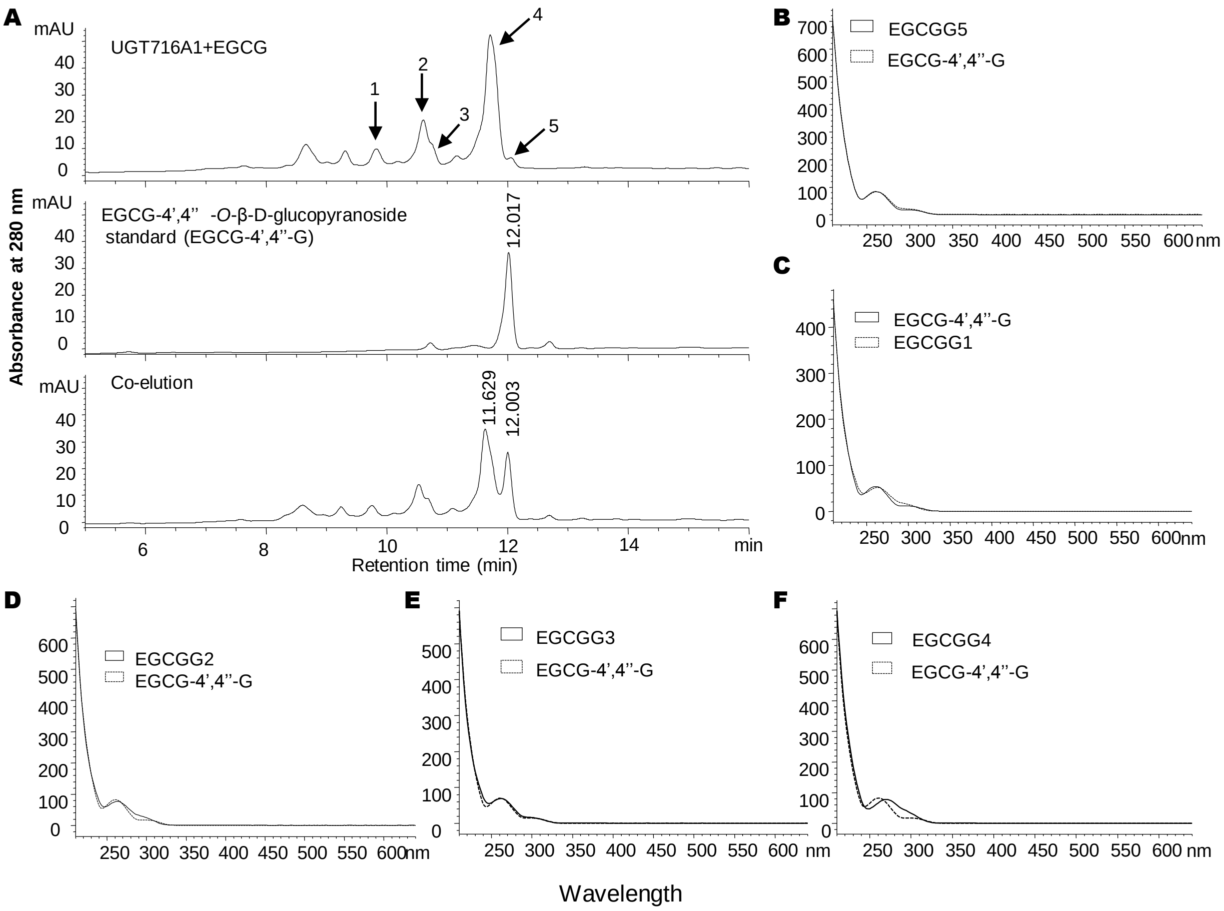

Figure S9. Comparison of the enzymatic products of UGT716A1 toward EGCG with authentic EGCG-4’,4”-Glu standard.

(A) HPLC chromatograms of enzymatic products from enzymatic assays of the recombinant UGT716A1 protein with EGCG as substrate (upper panel), EGCG-4’,4”-Glu authentic standard control (middle panel) and the co-elution of A and B (lower panel);

(B) UV spectrum of EGCGG5 in compared with the EGCG-4’,4”-Glu authentic standard;

(C-F) UV spectrum of EGCGG1, 2, 3, 4 in compared with the EGCG-4’,4”-Glu authentic standard.

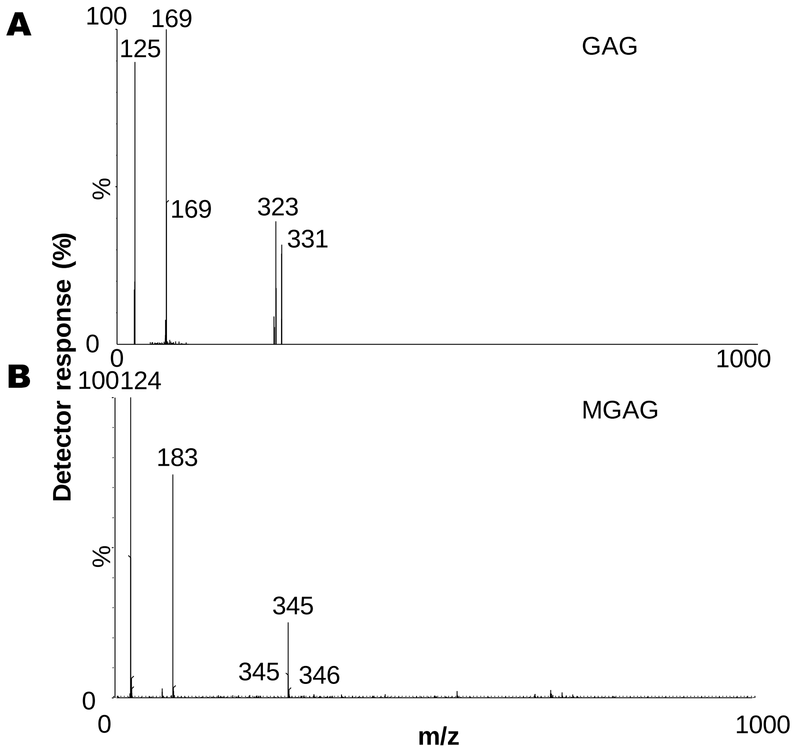

Figure S10. Mass spectra of the enzymatic products of recombinant UGT716A1 with (methyl)-gallic acid as substrates.

(A-B) Mass spectrum of the enzymatic products of the recombinant UGT716A1 protein with substrates of gallic acid (A) and methyl gallic acid (B).

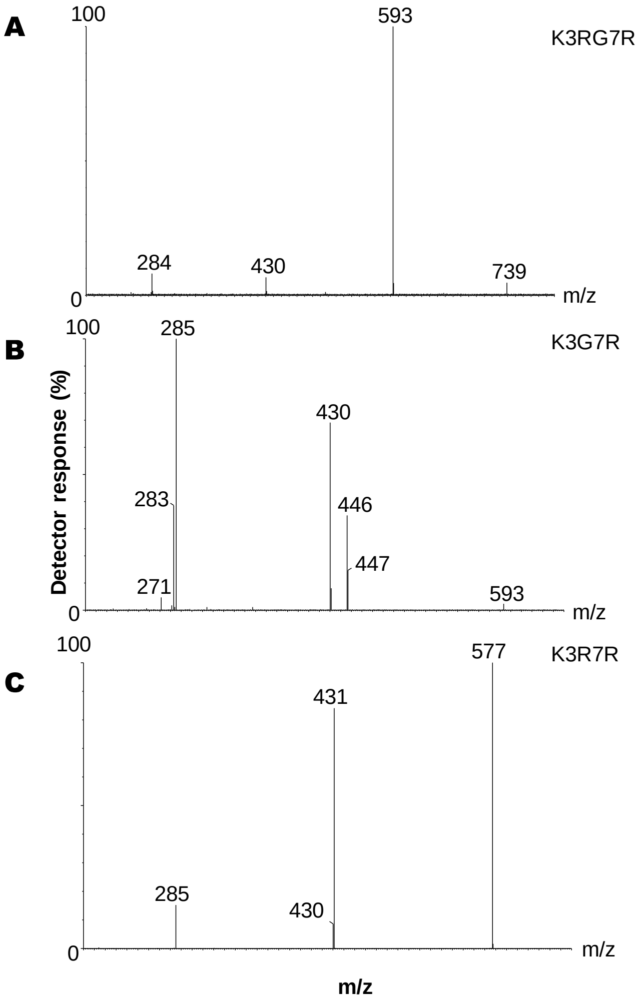

Figure S11. Mass spectra of the three major flavonols detected in *A. thaliana* seedlings.

(A) Mass spectrum of K3RG7R;

(B) Mass spectrum of K3G7R;

(C) Mass spectrum of K3R7R.

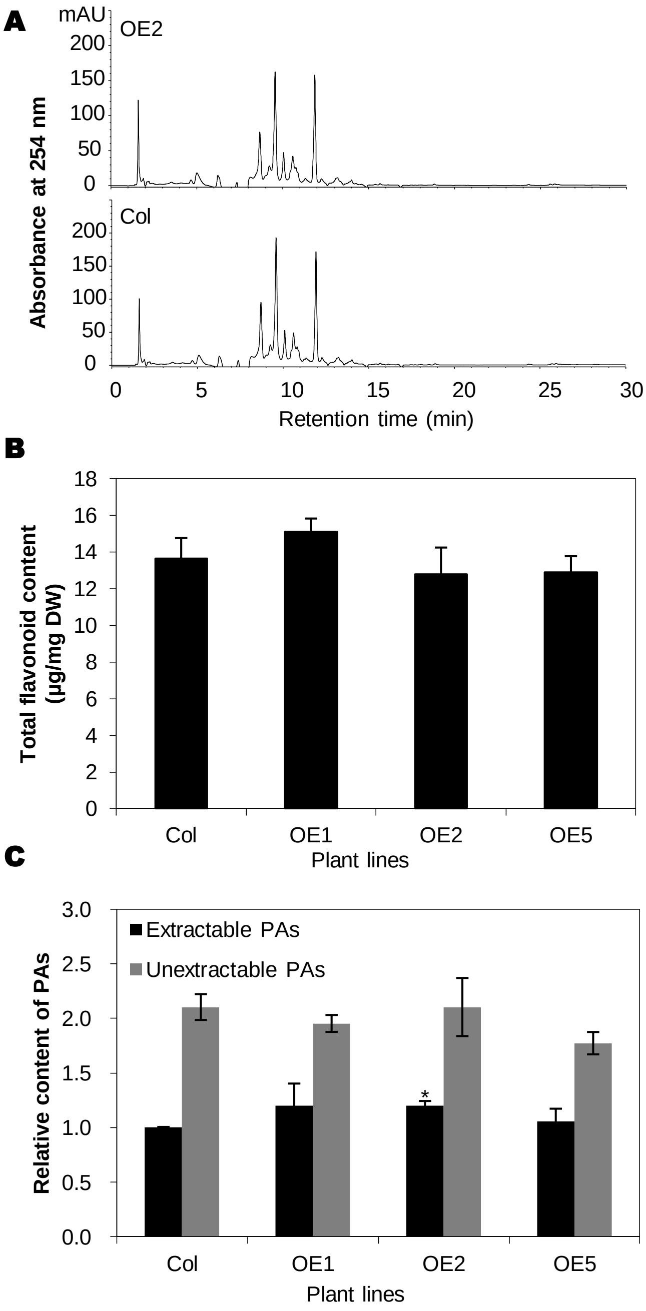

Figure S12. Analyses of flavonoids in transgenic *A. thaliana* over-expressing the *UGT716A1* gene.

(A) HPLC chromatograms of flavonoid profiles in seeds of transgenic line (OE2) and wild type control (Col);

(B) Total flavonoid content in seeds of transgenic lines and wild type control (Col);

(C) Relative proanthocyanidin content in seeds of transgenic lines and wild type control (Col).

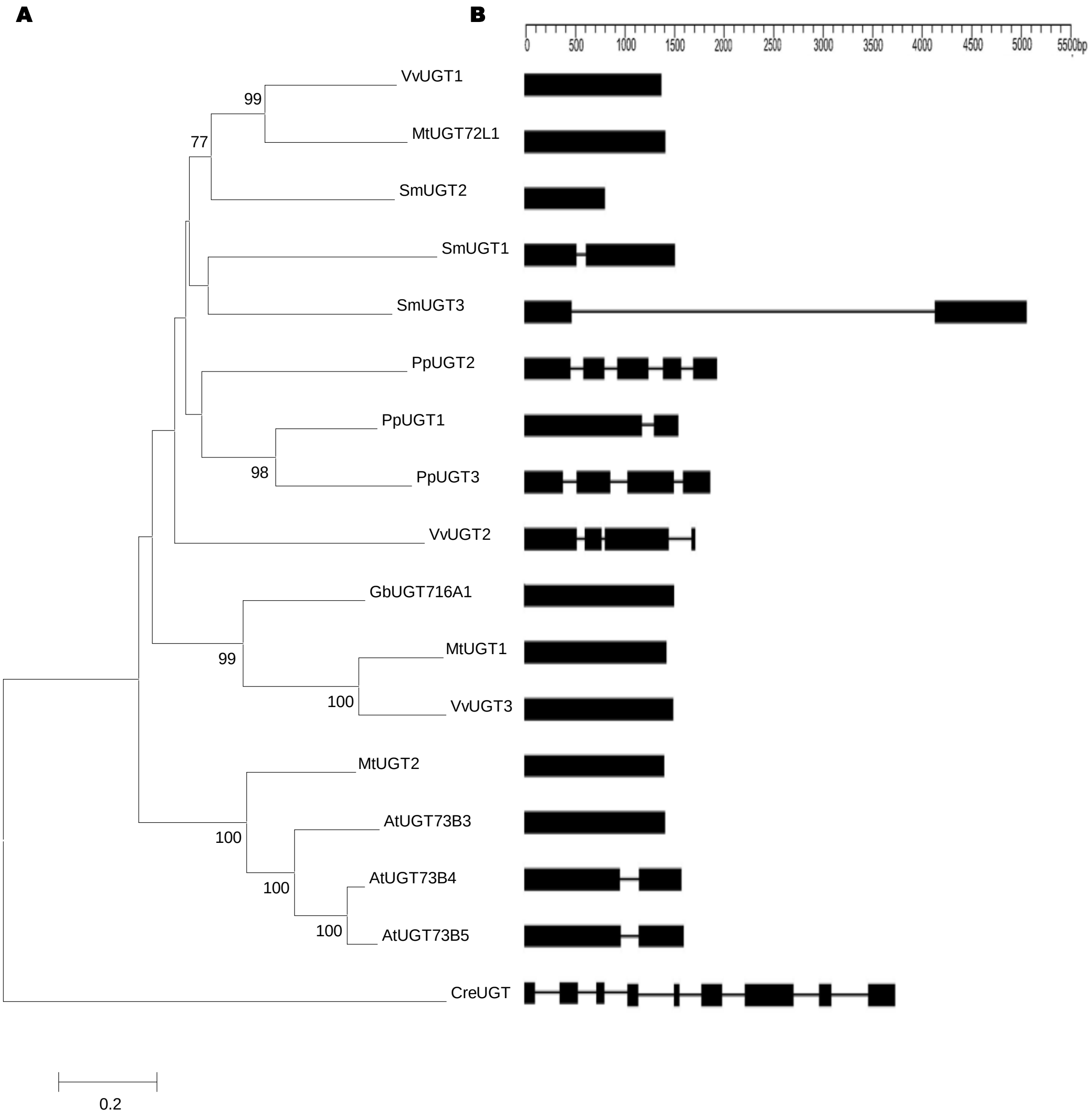

Figure S13. Phylogenetic analyses and gene structures of *UGT716A1* and its homologs.

(A) Phylogenetic analyses of UGT716A1 and its homology UGTs in other plant species. A neighbor-joining tree was constructed with Mega 6.0. Distance calculation was performed with Poisson correction. Branch lengths indicate the number of substitutions per site. Bootstrap analyses were performed with 1000 replicates and only values above 50% are shown. Sequences information are as follows: CreUGT, Cre07.g322884 (*Chlamydomonas reinhardtii*); SmUGT1, XP_002990186.1 (*Selaginella moellendorffii*); SmUGT2, XP_002987599.1 (*S. moellendorffii*); SmUGT3, XP_002971418.1 (*S. moellendorffii*); PpUGT1, XP_001760897.1 (*Physcomitrella patens*); PpUGT2, XP_001770048.1 (*P. patens*); PpUGT3,

XP_001778080.1 (*P. patens*); MtUGT1, XP_003612636.1 (*M. truncatula*); MtUGT2, XP_013456871 (*M. truncatula*); MtUGT72L1, ACC38470 (*M. truncatula*); VvUGT1, CBI34463.3 (*V. vinifera*); VvUGT2, XP_010650424.1 (*V. vinifera*); VvUGT3, XP_010664783.1 (*V. vinifera*); AtUGT73B3, Q8W491.1 (*A. thaliana*); AtUGT73B4, EFH60157.1 (*A. thaliana*); AtUGT73B5, Q9ZQG4.1 (*A. thaliana*).

(B) Gene structures of selected *UGT* genes. Extrons are shown in black box and introns are shown in line.

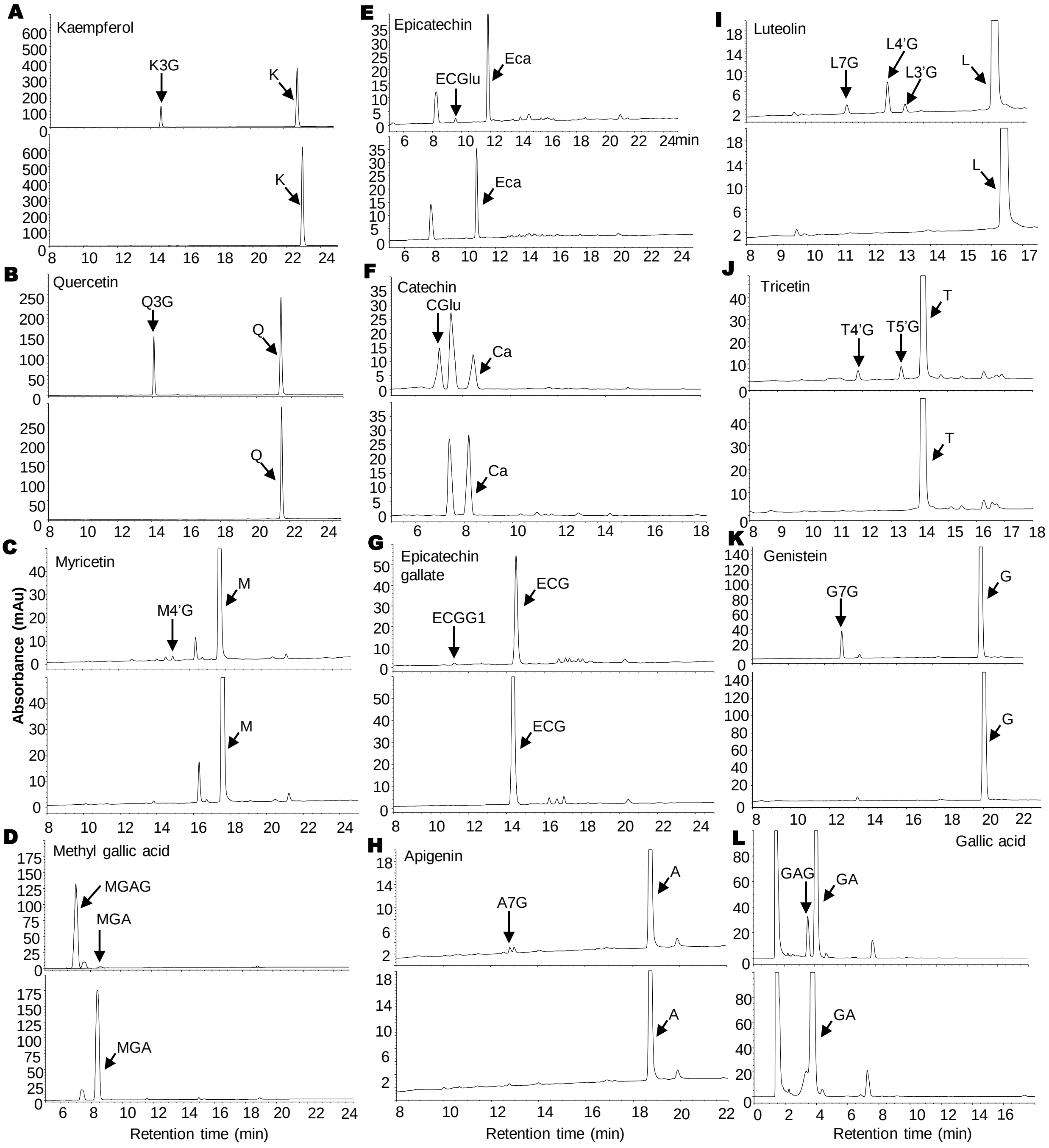
A
Kaempferol
K3G
K
B
Quercetin
Q3G
Q
C
Myricetin
M4'G
M
D
Methyl gallic acid
MGAG
MGA
E
Epicatechin
ECGlu
Eca
F
Catechin
CGlu
Ca
G
Epicatechin gallate
ECGG1
ECG
H
Apigenin
A7G
A
I
Luteolin
L7G
L4'G
L3'G
L
J
Tricetin
T4'G
T5'G
T
K
Genistein
G7G
G
L
Gallic acid
GAG
GA
Absorbance (mAu)
Retention time (min)
min

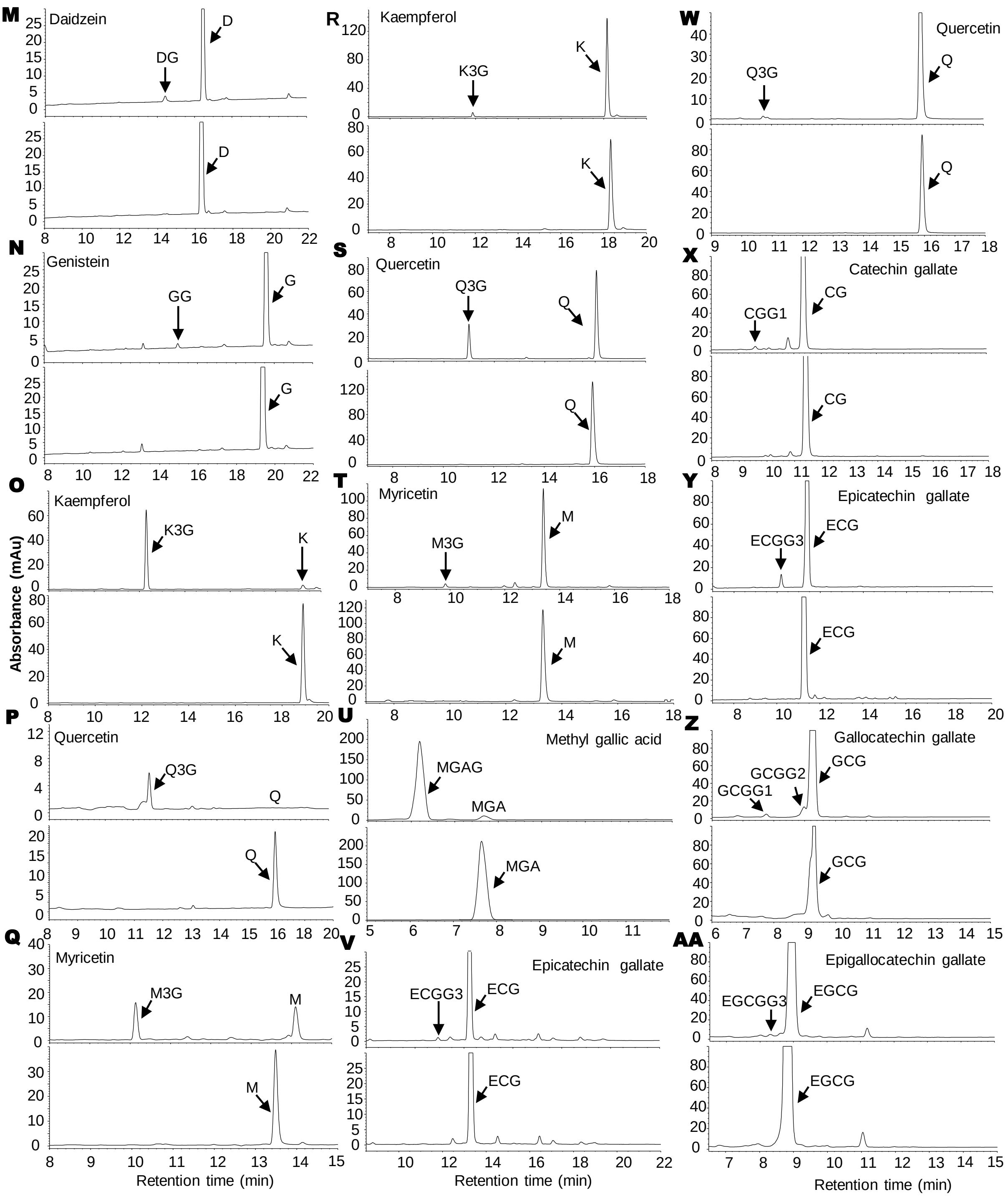

Figure S14. HPLC chromatograms of the enzymatic products with recombinant UGT proteins from different plant species.

(A-Ll) HPLC chromatography of recombinant PpUGT1 protein with various substrates: kaempferol (A), quercetin (B), myricetin (C), methyl gallic acid (D), epicatechin (E), catechin (F), epicatechin gallate (G), apigenin (H), luteolin (I), tricetin (J), genistein (K), and gallic acid (L);

(M-N) HPLC chromatograms of recombinant PpUGT2 protein with various substrates: daidzein (M) and genistein (N);

(O-Q) HPLC chromatograms of recombinant PpUGT3 protein with various substrates: kaempferol (O), quercetin (P), and myricetin (Q);

(R-V) HPLC chromatograms of recombinant VvUGT1 protein with various substrates: kaempferol (R), quercetin (S), myricetin (T), methyl gallic acid (U), and epicatechin gallate (V);

(W-AA) HPLC chromatogram of recombinant VvUGT3 protein with various substrates: quercetin (W), catechin gallate (X), epicatechin gallate (Y), gallocatechin gallate (Z), epigallocatechin gallate (AA).

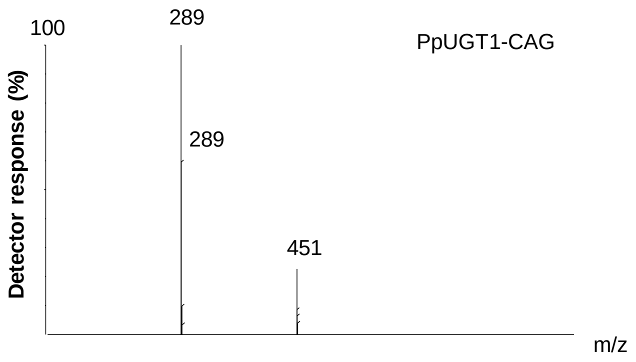

Figure S15. Mass spectra of the enzymatic product of recombinant PpUGT1 with catechin as substrate.

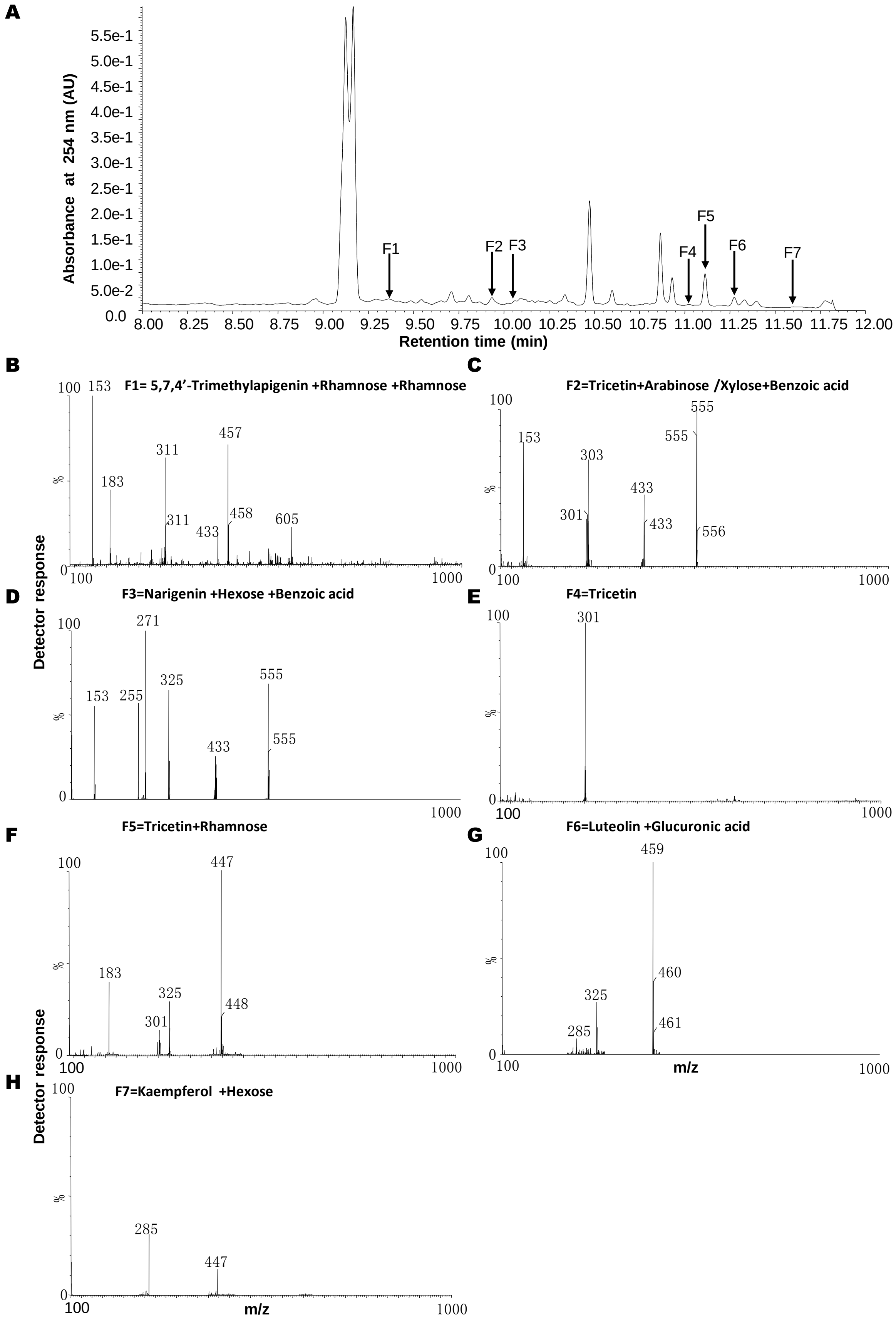

A
Absorbance at 254 nm (AU)
5.5e-1
5.0e-1
4.5e-1
4.0e-1
3.5e-1
3.0e-1
2.5e-1
2.0e-1
1.5e-1
1.0e-1
5.0e-2
0.0
8.00 8.25 8.50 8.75 9.00 9.25 9.50 9.75 10.00 10.25 10.50 10.75 11.00 11.25 11.50 11.75 12.00
Retention time (min)
F1 F2 F3 F4 F5 F6 F7
B
F1= 5,7,4'-Trimethylapigenin +Rhamnose +Rhamnose
153 183 311 311 433 457 458 605
C
F2=Tricetin+Arabinose /Xylose+Benzoic acid
153 301 303 433 433 555 555 556
D
F3=Narigenin +Hexose +Benzoic acid
153 255 271 325 433 555 555
E
F4=Tricetin
301
F
F5=Tricetin+Rhamnose
183 301 325 447 448
G
F6=Luteolin +Glucuronic acid
285 325 459 460 461
H
F7=Kaempferol +Hexose
285 447
Detector response
%
100
0
1000
m/z

Figure S16. Identification of putative flavonoid compounds in *P. patens* by UPLC/MS.

(A) HPLC chromatograms of putative flavonoid compounds identified in *P. patens* at the wavelength of 254 nm.

(B-H) Mass spectra of seven putative flavonoids labeled aglycones plus conjugates.
